# 2,2′‐Bipyridine‐Modified Tamoxifen: A Versatile Vector for Molybdacarboranes

**DOI:** 10.1002/cmdc.201900554

**Published:** 2019-11-18

**Authors:** Benedikt Schwarze, Sanja Jelača, Linda Welcke, Danijela Maksimović‐Ivanić, Sanja Mijatović, Evamarie Hey‐Hawkins

**Affiliations:** ^1^ Leipzig University Faculty of Chemistry and Mineralogy, Institute of Inorganic Chemistry Johannisallee 29 04103 Leipzig Germany; ^2^ University of Belgrade Department of Immunology, Institute for Biological Research “Siniša Stanković” – National Institute of Republic of Serbia Bul. Despota Stefana 142 11060 Belgrade Serbia

**Keywords:** breast cancer, molybdacarborane, tamoxifen, nitric oxide (NO), nanoparticles

## Abstract

Investigations on the antitumor activity of metallacarboranes are sparse in the literature and limited to a handful of ruthena‐ and molybdacarboranes. In this study, the molybdacarborane fragment [3‐(CO)_2_‐*closo*‐3,1,2‐MoC_2_B_9_H_11_] was combined with a vector molecule, inspired by the well‐known drug tamoxifen or 4,4′‐dihydroxytamoxifen (TAM‐diOH). The molybdacarborane derivative [3,3‐{4‐[1,1‐bis(4‐hydroxyphenyl)but‐1‐en‐2‐yl]‐2,2′‐bipyridine‐κ^2^ *N*,*N*′}‐3‐(CO)_2_‐*closo*‐3,1,2‐MoC_2_B_9_H_11_] (**10**), as well as the ligand itself 4‐[1,1‐bis(4‐hydroxyphenyl)but‐1‐en‐2‐yl]‐2,2′‐bipyridine (**6**) showed cytotoxic activities in the low micromolar range against breast adenocarcinoma (MDA‐MB‐231, MDA‐MB‐361 and MCF‐7), human glioblastoma (LN‐229) and human glioma (U‐251) cell lines. In addition, compounds **6** and **10** were found to induce senescence and cytodestructive autophagy, lower ROS/RNS levels, but only the molybdacarborane **10** induced a strong increase of nitric oxide (NO) concentration in the MCF‐7 cells.

## Graphical abstract

**Privileged structures**: Applying this drug design concept on the well‐known anticancer drug tamoxifen leads to its 2,2′‐bipyridine‐modified derivative, which is a vector for molybdacarboranes. The biological activity profile contrasts with those of other organic or inorganic selective estrogen receptor modulators: whereas the ROS/RNS levels are decreased, the NO levels increase. The ability to form nanoparticles with bovine serum albumin opens the way to modern nanomedicine.

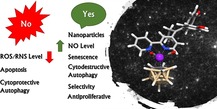

## Introduction

Medicinal chemistry is still dominated by purely organic molecules, but bioinorganic chemistry is becoming more and more important and several successful examples of metal‐based drugs have already been reported (see special issue on *Metals in Medicine* in ACS Chemical Reviews).^1^ However, the available biological data for molybdenum(II) complexes of the type [Mo(R)(L)X] (where R=(η^3^‐C_3_H_5_)^−^, (η^5^‐C_5_H_5_)^−^, (η^5^‐C_9_H_7_)^−^; L=1,10‐phenanthroline, 2,2′‐bipyridine, (CO)_2_, (MeCN)_2_; X=Cl^−^, BF_4_^−^) are very limited, and only a few studies were reported until now.^2^ On the other hand, commercially available boron‐based drugs are still rare, and mainly employed in boron neutron capture therapy (BNCT).^3^ Unlike hydrocarbons, boranes and carboranes readily form clusters in a large variety of 3D geometric shapes. The icosahedral *closo*‐dicarbadodecaboranes or carboranes are highly interesting, due to their hydrophobicity, which is beneficial for transport across the blood‐brain barrier (BBB), their inorganic nature preventing enzymatic degradation, their inherent low toxicity lowering side effects, their high boron content for BNCT, the variety of possible substitution patterns employing the three isomers (*ortho‐*, *meta‐*, *para*‐carborane) and their 3D aromatic structures can be employed as bulky phenyl or cyclopentadienyl analogues.^4^ Thus, a deboronation (i. e. formal loss of a B^+^ unit) of the *closo*‐C_2_B_10_H_12_ cluster and deprotonation, the so‐called dicarbollide, [C_2_B_9_H_11_]^2−^, is obtained and can be employed in the preparation of a large variety of transition metal complexes resulting in full‐, mixed‐ or half‐sandwich structures.^5^ Potential applications of the full‐sandwich cobalt bis(dicarbollide) ([3,3′‐Co(1,2‐C_2_B_9_H_11_)_2_]^−^, COSAN) derivatives in medicine are well investigated,^6^, ^7^ but not as a pharmacophore. Also cytotoxic/cytostatic icosahedral mixed‐sandwich metallacarboranes, e.g. [3‐(η^6^‐arene)‐3,1,2‐RuC_2_B_9_H_11_], have been reported.^8^, ^9^ Other half‐ and mixed‐sandwich complexes with potential application in medicine have been summarized in the literature.^4^ One advantage of half‐sandwich metallacarboranes is the incorporation of biologically relevant ligands in the coordination sphere of the metals, as in the present study, where [3‐(2,2′‐bipyridine‐κ^2^ *N*,*N*′)‐3‐(CO)_2_‐*closo*‐3,1,2‐MoC_2_B_9_H_11_] (**i**) was equipped with a vector molecule which was inspired by the well‐known tamoxifen. Further examples were reported by Causey et al.,^10^ Hawkins et al.,^11^ Pruitt et al.^12^ and Louie et al.^13^ who used stable and hydrophobic rhenacarboranes including the [Re(L)(CO)_2_] fragment (L=2,2′‐bipyridine derivative or [NO]^+^) as imaging agents.

**Scheme 1:**
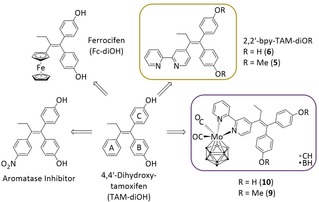
Bioisosteric replacement approach: from TAM‐diOH to ferrocifen (Fc‐diOH), an aromatase inhibitor,^21^ 2,2′‐bpy‐TAM‐diOR (R=Me (**5**), H (**6**)), [[3,3‐{4‐[1,1‐bis(4‐methoxyphenyl)but‐1‐en‐2‐yl]‐2,2′‐bipyridine‐κ^2^ *N,N′*}‐3‐(CO)_2_‐*closo*‐3,1,2‐MoC_2_B_9_H_11_] (**9**) or [3,3‐{4‐[1,1‐bis(4‐hydroxyphenyl)but‐1‐en‐2‐yl]‐2,2′‐bipyridine‐κ^2^ *N,N′*}‐3‐(CO)_2_‐*closo*‐3,1,2‐MoC_2_B_9_H_11_] (**10**).

Nuclear receptor ligands using highly hydrophobic icosahedral carborane clusters in structures like estradiol,^14^ testosterone^15^ or selective estrogen receptor modulators (SERMs)^16^ were already reported. Also, a carborane‐containing tamoxifen derivative, namely boroxifen, was synthesized, which showed, however, limited benefits.^17^ Employing tamoxifen as lead structure is promising, because it exhibits estrogenic/antiestrogenic activity as well as cytotoxic properties,^18^ and is also active in prevention of osteoporosis in postmenopausal women with early‐stage breast cancer^19^ and in the protection of cardiovascular functions.^20^ Furthermore, its mode of action can easily be modified by small changes; e. g. an “A” ring substitution (Scheme 1) or modification with a nitro group in *para* position leads to aromatase inhibition (the enzyme producing estrogen).^21^

One further prominent example is the ferrocene‐containing derivative ferrocifen (Fc‐diOH) (Scheme 1), which was reported and extensively biologically tested by Jaouen *et al.*. Ferrocifen showed redox chemistry useful for the generation of reactive oxygen (ROS) and nitrogen species (RNS) via Fenton chemistry induced by the ferrocene unit^22^, ^23^, ^24^ and high activity towards hormone‐dependent (e. g. MCF‐7) and “hormone‐independent” (e. g. MDA‐MB‐231) cell lines.^25^ Also a few other metal complex‐based SERMs are known.^26^ Even though estrogenic activity (agonist or antagonist) was proven for ferrocifen, this effect is only dominant at nanomolar concentrations, whereby other modes of action, like induction of senescence (ca. 10^−7^ m), apoptosis (ca. 10^−6^–10^−5^ m) or Fenton chemistry (ca. 10^−5^–10^−4^ m) become the predominant effects at higher concentrations of ferrocifen *in vitro*.^27^ However, so far, no metallacarborane was combined with the tamoxifen lead structure. Therefore, the “A” ring in 1,1‐bis(4‐hydroxyphenyl)‐2‐phenylbut‐1‐ene (TAM‐diOH) was replaced with a well‐known chelating moiety (2,2′‐bipyridine), being able to coordinate a variety of potential biologically active transition metals (be it a pharmacophore or a traceable element, etc.), with the goal to obtain novel potential SERMs (Scheme 1).

We here report on the combination of this novel 2,2′‐bpy‐TAM‐diOH vector system with half‐sandwich molybdacarboranes.^28^ For antitumor studies, a formulation strategy using bovine serum albumin (BSA) that was developed by us was applied, which led to a substantial increase in the biological activity against the MCF‐7 breast cancer cell line for the new pharmacophore [3‐(2,2′‐bipyridine‐κ^2^ *N*,*N*′)‐3‐(CO)_2_‐*closo*‐3,1,2‐MoC_2_B_9_H_11_] (**i**).^29^

## Results and Discussion

### Syntheses and Characterization

**Scheme 2:**
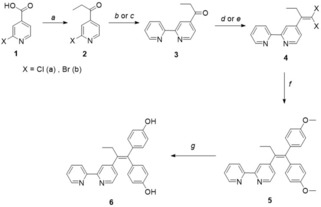
Synthetic route yielding the organic vector incorporating the chelating 2,2′‐bipyridine unit. (a) EtMgBr (2.2 equiv.) in THF, −40 °C, 4 h; (b) reflux conditions: 2‐(tributylstannyl)pyridine (1.1 equiv.), [Pd(PPh_3_)_4_] (10 mol%), CsF (2.2 equiv.), CuI (16 mol%) in DMF, 120 °C, 3 d; (c) microwave conditions: 2‐(tributylstannyl)pyridine (1.1 equiv.), [Pd(PPh_3_)_4_] (10 mol%), CsF (2.2 equiv.), CuI (16 mol%) in DMF, 140 °C, 16 h; (d) CBr_4_, P(O^i^Pr)_3_ in DCM, 0 °C to 40 °C, 4 d; (e) CCl_4_, PPh_3_ in toluene, 110 °C, 3 d; (f) 4‐methoxyphenylboronic acid (5.0 equiv.), [Pd(PPh_3_)_4_] (10 mol%), Na_2_CO_3_ (5.0 equiv.) in 1,4‐dioxane/H_2_O (4 : 1), 100 °C, 4 d; (g) BBr_3_ in DCM, −60 °C to rt, 12 h.

The synthesis of the 2,2′‐bipyridine‐substituted tamoxifen derivative **6** (Scheme 2) via the facile McMurry coupling was not successful, probably due to the electron‐poor 2,2′‐bipyridine unit, which renders the ketone in compound **3** less reactive in this type of olefination reaction, unlike the electron‐rich ferrocene derivative in the synthesis of ferrocifen.^30^ Equally unsuccessful was the use of **3** in a Wittig‐Horner‐type reaction. Therefore, ligand **6** was constructed stepwise starting from the commercially available 2‐halo‐isonicotinic acid (**1** **a**,**b**), which was reduced to 1‐(2‐halopyridin‐4‐yl)propan‐1‐one (**2** **a**,**b**) with ethylmagnesium bromide in tetrahydrofuran (THF) at −40 °C (Scheme 2).^31^ A [Pd(PPh_3_)_4_]‐catalyzed Stille coupling reaction resulted in the asymmetric 2,2′‐bipyridine derivative (**3**) in moderate to good yields (gram scale 56 %, small scale 69 %). A Horner‐Wadsworth‐Emmons modification for the Ramirez *gem*‐dibromoolefination gave compound **4** in good yield (78 %).^32^ A [Pd(PPh_3_)_4_]‐catalyzed Suzuki coupling afforded compound **5** in good yield (76 %). Demethylation of the aryl‐methyl ether **5** with BBr_3_ resulted in the final ligand **6**.^33^ The conditions for the optimization of both Pd^0^‐catalyzed coupling reactions are given in the Supporting Information (Tables S1 and S2, SI). Compounds **3**–**6** were fully characterized by NMR, infrared spectroscopy, mass spectrometry, and elemental analysis; the solid‐state molecular structure of **6** was also confirmed by single crystal X‐ray diffraction methods (Figure S1, SI). After obtaining the new ligands, coordination studies were performed with [NEt_4_][3‐(η^3^‐C_3_H_5_)‐3‐(CO)_2_‐*closo*‐3,1,2‐MoC_2_B_9_H_11_] (**7**).

**Scheme 3:**
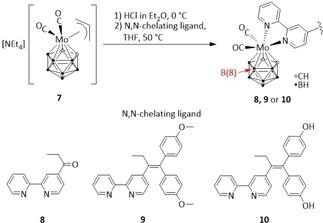
Synthesis of the molybdacarboranes **8**, **9** and **10** via protonation of [NEt_4_][3‐(η^3^‐C_3_H_5_)‐3‐(CO)_2_‐*closo*‐3,1,2‐MoC_2_B_9_H_11_] (**7**) and reaction with N,N‐chelating ligands **3**, **5** or **6**. Both coordination isomers are formed for **8**, **9** and **10**.

In **7**, the acid‐labile allyl ligand at molybdenum(II) was released upon protonation and then replaced with the respective N,N‐chelating ligand **3**, **5** and **6** to give the molybdacarborane complexes **8**, **9** and **10**, respectively, according to Schwarze *et al*. (Scheme 3).^28^ Unlike the molybdacarboranes bearing a simple 2,2′‐bipyridine or 1,10‐phenanthroline ligand,^28^ complexes **8**–**10** could be purified by flash column chromatography on silica gel in low to moderate yields (18–35 %). These low yields are most likely related to a side reaction which was not observed for the simple N,N‐chelating ligands (e. g. 2,2′‐bipyridine or 1,10‐phenanthroline).^28^ During the purification process, more than one violet band could be observed for every complexation reaction. Exemplarily for **9**, the second violet fraction was collected in sufficient purity and identified by ^1^H, ^13^C{^1^H}, ^11^B{^1^H} and ^11^B NMR spectroscopy, HR‐ESI mass spectrometry and diffraction methods as a chlorine B(8)‐substituted derivative of **9** (see SI), where the respective position in the carborane ligand was activated for EINS‐type (electrophile induced nucleophilic substitution) reactions (**9** **b**, Figure S2, SI).^34^ This one‐pot two‐step reaction (proton‐mediated ligand exchange and EINS‐type reaction at the dicarbollide) was attempted before without success for complexes with the 2,2′‐bipyridine and 1,10‐phenanthroline ligand, [3‐(L‐κ^2^ *N*,*N*′)‐3‐(CO)_2_‐*closo*‐3,1,2‐MoC_2_B_9_H_11_], with L=2,2′‐bipyridine or 1,10‐phenanthroline, using different strong acids and solvents.^28^ Further studies for employing the chloride in carborane substitution chemistry are in progress.

The color of all the ligands ranges from colorless to pale yellow. On coordination to molybdenum(II), a color change to deep purple is observed for complexes **8**–**10** (for UV‐Vis spectra of **6** and **10** see Figure S16, SI) indicating metal‐to‐ligand (MLCT) and ligand‐to‐metal charge transfer (LMCT) (with L=**3**, **5** or **6**).^28^ In the infrared spectra, typical vibrations of all functional groups are observed, e. g. ν(BH) (2595–2480 cm^−1^) and ν(CO) (1955–1874 cm^−1^) vibrations, which are unique in biological systems and could, therefore, be used for label‐free imaging (first attempts have been reported for [3‐(2,2′‐bipyridine‐κ^2^ *N*,*N*′)‐3‐(CO)_2_‐*closo*‐3,1,2‐MoC_2_B_9_H_11_].^29^ In the ^1^H NMR spectra of the complexes **8**, **9** and **10**, there is a clear coordination shift observed compared to the chemical shifts of the ligands **3**, **5** and **6** (Figure S3–S5, SI). The CH_cluster_ signals split into two broad singlets due to the asymmetric N,N‐chelating ligand. Importantly, the coordination of **3**, **5** and **6** at molybdenum(II) always generates two isomers, which cannot be distinguished via NMR spectroscopy. The carbonyl groups are observed as two slightly different signals at 255.3 and 254.5 ppm (**8**, CD_2_Cl_2_), 256.3 and 255.5 ppm (**9**, CDCl_3_) or 260.3 and 260.0 ppm (**10**, CD_3_CN) in the ^13^C{^1^H} NMR spectra of the respective complexes according to the C_1_ symmetry of the whole molecule.

Obtaining HR‐ESI mass spectra of molybdacarboranes is challenging, since they cannot be ionized easily and are either detectable in negative or positive mode. Additionally, a strong clustering effect can be observed for complexes **8**–**10**. For illustration, one typical high‐resolution ESI mass spectrum in the negative mode is depicted in the supporting information (Figure S18, SI), showing an agglomeration pattern, where the unidentifiable agglomerate X (m/z=1965.6786) loses five times a fragment [M−2H]^2−^. This effect can be minimized through dissolution of the sample immediately before injection into the ESI‐MS instrument. This is a nice demonstration of the potential agglomeration properties of metallacarboranes in solution.

### Bioanalytical Measurements

For *in vitro* cell culture tests, the stock solutions of sparingly water‐soluble compounds are typically prepared in DMSO (ethanol or methanol are good alternatives) and stored below +4 °C. For that purpose, the chemical stability of **3**, **5**, **6** and **8**–**10** was tested in a solution of water‐containing DMSO‐d_6_ in air for at least 36 days (**3**, **5**, **6**, **8**, **9**) and for 14 days for complex **10**. ^1^H and ^11^B{^1^H} NMR spectra revealed that the ligands **3**, **5** and **6** can be stored in a DMSO stock solution for at least one month without decomposition, and the molybdacarboranes for 14 days up to one month with minor decomposition (where the decomposition products are the free ligands **3**, **5** or **6**, the *nido*‐carborane ([*nido*‐C_2_B_9_H_12_]^−^) and most likely a molybdenum species in higher oxidation states) (see Figure S6–S14). Recently, we introduced a formulation procedure for sparingly water‐soluble metallacarboranes, using fatty acids‐ and magnesium‐free bovine serum albumin (BSA_noMg_) in a 1 : 10 molar ratio, which led to a significant improvement of the reproducibility, but also the cytotoxicity against MCF‐7 breast cancer cells.^29^ Here, the biological activity of molybdacarboranes (**8**–**10**), as well as ligands **3**, **5** and **6**, and literature‐known reference compounds (TAM‐diOH and Fc‐diOH) was evaluated employing this formulation procedure to ensure comparability.

To understand the self‐assembling behavior of metallacarboranes/BSA_noMg_ co‐assemblies, exemplarily ligand **6** and complex **10** were investigated in phosphate‐buffered saline (PBS)/DMSO mixtures at physiological pH (pH 7.4) by UV‐Vis, fluorescence and Rayleigh light scattering (RLS) spectroscopy, as well as Nanoparticle Tracking Analysis (NTA).^9^, ^29^, ^35^

For the fluorescence spectra, three excitation wavelengths were chosen (λ_exc_=280, 295 and 320 nm) and the investigations were performed with two site markers, namely warfarin and ibuprofen, binding selectively to Sudlow's site I or Sudlow's site II, respectively. The same systems were investigated by UV‐Vis spectroscopy in parallel. Our measurements revealed that **6** binds to both, Sudlow's site I and II (Figure S15, SI).

Remarkably, the fluorescence intensity at λ_exc_=280 nm is quenched to the same extent when **6** only is mixed with BSA_noMg_ or together with ibuprofen, indicating that the binding strength is in the same order of magnitude as for the site marker. The situation is slightly different for **10**, which binds to Sudlow's site II, but the fluorescence is quenched more in the ternary system together with BSA_noMg_ and ibuprofen. The fluorescence intensity is quenched more when **10** is added first, and ibuprofen afterwards, which might imply a weak cooperative effect upon binding of both components (Figure S15, SI). Evidently, there is a strong cooperative effect of both **6** and **10** with warfarin (at the excitation wavelengths λ_exc_=295 and 320 nm), which is independent from the order of addition. Also, tamoxifen acts differently, having a negative allosteric effect on warfarin–HSA binding.^36^ These findings are in contrast with the ones for the molybdacarborane [3‐(2,2′‐bipyridine‐κ^2^ *N*,*N*′)‐3‐(CO)_2_‐*closo*‐3,1,2‐MoC_2_B_9_H_11_] (**i**), which seems to bind stronger to Sudlow's site I than to site II, without any interaction being detected between warfarin and **i**.^29^ The absorption spectra of the ternary systems (BSA‐site marker‐drug, Figure S16, SI) show the same overall trend concerning binding events as the fluorescence spectra. Notably, the UV‐Vis spectrum of **10** in PBS/DMSO mixture is essentially the same as for **i**; therefore, similar electronic structures can be assumed.^28^ The absorption spectrum suggests that the solubility of **6** is improved on binding to BSA_noMg_, because the baseline shift, due to scattering, is suppressed. This was confirmed by measuring Rayleigh Light Scattering (RLS), which showed a decrease of scattering aggregates, in an analogous way as we recently reported (Figure S17 left, SI).^29^ For **10**, the binding to BSA (1 : 1) seems to increase the scattering according to RLS (Figure S17 right, SI).

**Figure 1:**
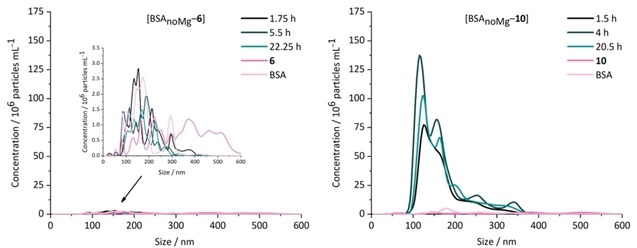
Size distribution of **6** (left) and **10** (right) in PBS/DMSO mixtures from NTA measurements. Ratio BSA_noMg_:**6** or **10** was 10 : 1. [**6**]=[**10**]=20 μm, [BSA]=200 μm. The dilution factors are adjusted to 11x for each sample. Samples were measured 1.5–22.25 h after preparation. The respective blanks (BSA_noMg_ in PBS/DMSO) are measured after 2.2 h (**6**) or 2.4 h (**10**); **6** in PBS/DMSO after 2 h, **10** in PBS/DMSO after 0.5 h. Standard deviation (SD) for particle concentration is ±1.30–3.68×10^6^ (samples regarding **6**) and ±2.68×10^6^–3.88×10^7^ (samples regarding **10**) particles mL^−1^, for particle size ±4–18 nm.

Nanoparticle Tracking Analysis (NTA) measurements were performed in a PBS/DMSO mixture exemplarily for **6** and **10**, with and without the addition of fatty acids‐ and magnesium‐free bovine serum albumin (BSA_noMg_) in a 10 : 1 ratio. A sharp increase in particle concentration is observed when the formulation protocol is applied for molybdacarborane **10** with bimodal distribution (after deconvolution) and relatively low polydispersity (Figure 1, right) being stable over 20 h, supporting the findings from previous studies.^29^ Interestingly, when the aggregation behavior of only the ligand **6** is investigated, no such effect is observed. Indeed, the very broad size distribution of particles of **6** in PBS/DMSO is transformed into a size distribution that resembles BSA in PBS/DMSO (Figure 1, left) implying that **6** is bound to BSA (see also spectroscopic data: Figure S15–S16, SI), but does not induce agglomeration as metallacarboranes do. Thus, these findings are a strong evidence that metallacarboranes form nano‐sized aggregates with BSA_noMg_, which cannot be observed for organic hormone‐like structures, even though binding to BSA_noMg_ was proven for **6** via UV‐Vis and fluorescence spectroscopy.

### *In vitro* Cell Colorimetric Assays

After we found evidence of cytotoxic activity of [3‐(2,2′‐bipyridine‐κ^2^ *N*,*N*′)‐3‐(CO)_2_‐*closo*‐3,1,2‐MoC_2_B_9_H_11_] (**i**) against MCF‐7 cells,^29^ we wanted to expand our studies to other molybdacarboranes with N,N‐chelating ligands and a larger panel of cell lines, i. e. three breast cancer adenocarcinomas (MDA‐MB‐231, MDA‐MB‐361 and MCF‐7), human glioblastoma (LN‐229) and human glioma (U‐251) cell lines, plus mouse macrophages (Mf), as an example for non‐malignant immune cells (Table 1 and Figures S19 and S20, SI). Cells were exposed to **3**, **6** and **8**–**10** for 72 h, after which 3‐(4,5‐dimethylthiazol‐2‐yl)‐2,5‐diphenyltetrazolium bromide (MTT) and crystal violet (CV) cell viability assays were performed.

**Table 1:** IC_50_ values for **3**, **6**, **8–10** from MTT and CV cell viability assays. Standard deviations for each IC_50_ value are given.

		IC_50_ [μm]					
		Cells					
Compound	Assay	MDA‐MB‐231	MDA‐MB‐361	MCF‐7	U‐251	LN‐229	Mf
**3**	MTT	42.0±3.4	78.1±3.8	43.0±2.9	38.0±3.0	47.2±0.9	–^[a]^
	CV	38.1±3.5	70.5±5.2	41.3±2.2	44.1±1.6	54.9±4.1	>100
**6**	MTT	2.2±0.2	2.4±0.2	1.8±0.4	15.2±0.6	15.8±2.0	–^[a]^
	CV	2.5±0.2	2.8±0.3	2.1±0.6	21.0±3.5	17.7±1.0	>100
**8**	MTT	>100	71.0±4.4	34.4±1.7	97.4±3.7	>100	–^[a]^
	CV	>100	75.7±5.0	27.9±3.5	55.8±5.1	>100	>100
**9**	MTT	38.2±1.4	18.3±1.4	6.0±0.6	17.2±0.6	21.9±1.6	–^[a]^
	CV	30.8±2.8	18.1±2.4	6.6±0.9	17.4±1.0	25.8±1.7	>100
**10**	MTT	17.8±2.0	4.0±0.4	9.3±0.5	30.1±2.8	38.0±0.9	–^[a]^
	CV	18.5±1.1	4.5±0.6	5.0±0.7	32.7±3.8	41.0±3.8	>100
TAM‐diOH	MTT	15.4±0.6	24.5±3.6	24.3±1.6	>100	>100	–^[a]^
	CV	16.8±1.3	33.9±1.3	25.3±2.4	>100	>100	97.1±4.2
Fc‐diOH	MTT	1.0±0.1	1.5±0.3	1.0±0.1	1.4±0.2	3.1±0.2	–^[a]^
	CV	0.9±0.1	2.3±0.2	1.0±0.1	1.7±0.1	2.6±0.3	20.6±0.6

All experiments were done using BSA_noMg_ formulation strategy (BSA/small molecule (10 : 1)), before dilution with cell culture medium. Cell viability is expressed as percentage (%) relative to control and presented as mean ± SD of three independent experiments performed in triplicate. [a] “–“ means *not tested*. Only minor discrepancies in the calculated IC_50_ values obtained from the two assays (MTT or CV) were found for most of the tested compounds.

As we found an improvement of the cytotoxicity for **i** upon formulation with BSA_noMg_ in a 1 : 10 ratio (compound:BSA_noMg_) in previous studies, we performed all studies presented here with the mentioned formulation protocol, also for the non‐metallacarborane compounds **3** and **6**, as well as the two reference compounds 1,1‐bis(4‐hydroxyphenyl)‐2‐phenylbut‐1‐ene (TAM‐diOH) and ferrocifen (Fc‐diOH). Under the applied conditions of the biological evaluation, **5** was not suitable for testing due to precipitation from the solution, and **9** **b** could not be obtained in sufficient purity. The results revealed that ligand **3** and the corresponding metallacarborane **8** have moderate to low cytotoxic activity on the studied cell lines. In all cases, except for the MCF‐7 cells, the IC_50_ values for molybdacarborane complexes are slightly higher than for the respective ligands alone. Complex **9**, bearing a vector which is closer to the lead structure of TAM‐diOH, performs expectedly better, especially against the MCF‐7 cell line (Table 1). Remarkably, also the human glioblastoma cells (LN‐229) and human glioma cells (U‐251) were sensitive towards treatment with **9** in the low/moderate micromolar range. The couple **6** and **10** showed activity against all tested cell lines in the low micromolar range (Table 1). Worth highlighting is that the substitution of the phenyl ring “A” in TAM‐diOH by a chelating 2,2′‐bipyridine unit does not only improve the anticancer activity for all tested breast cancer cell lines, but also provides cytotoxic activity against the very aggressive primary malignant glioblastoma cells (LN‐229) and the invasive, malignant human glioma cells (U‐251). The anticancer activity for Fc‐diOH could be reproduced also with the BSA_noMg_ formulation protocol.^37^ In general, the IC_50_ values for Fc‐diOH against all malignant cell lines are lower than for the other tested compounds; however, **6**, **9** and **10** come close to the same activity range, depending on the cell line. Remarkably, the absolute toxicity of Fc‐diOH against healthy immune cells (mouse macrophages, Mf) is considerably high (20.6±0.6 μm) compared to **3**, **6** and **8**–**10** (>100 μm) (Table 1 and Figure S20, SI). This issue has already been reported for ferrocifen‐type anticancer drugs, showing an analogous high absolute toxicity (10 μm) against healthy brain tissue *in vivo*.^38^ However, relatively seen the preferential selectivity towards tumor cells is generally higher for Fc‐diOH (considering the experimental cut‐off at 100 μm). This, however, does not extend to **6** regarding all breast cancer cell lines and for **10** in the cases of the MDA‐MB‐361 and MCF‐7 cell lines.

Western blot analysis was performed to assess the estrogen receptor status (see Figure S21, SI), However, it seems that the anticancer potential of our newly designed compounds is not in correlation with estrogen receptor expression, under the applied concentrations, indicating the existence of other targets for these molecules.

### Flow Cytometry

To evaluate a possible mode of action of the new compounds, **6** and **10** were selected for further analysis on the MCF‐7 cell line by flow cytometry. Fc‐diOH was also tested, as reference, for comparison. All following investigations were performed applying the BSA_noMg_ formulation protocol.

**Figure 2:**
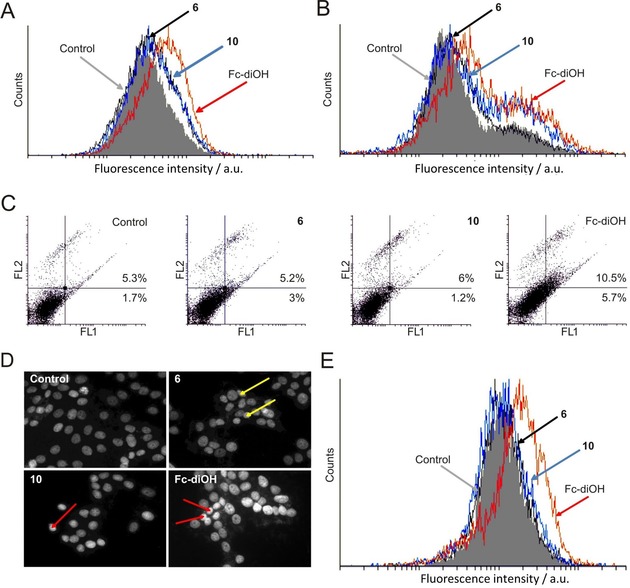
Flow cytometry analysis of MCF‐7 cells exposed (72 h) to an IC_50_ dose of **6**, **10** or Fc‐diOH, respectively, applying the BSA_noMg_ formulation procedure. (**A**) CFSE staining, fluorescence intensity from the FL1 channel (green emission); (**B**) senescence‐associated (SA) β‐galactosidase staining, fluorescence intensity from the FL1 channel; (**C**) AnnV/PI double staining, fluorescence intensity from FL2 (y axis, orange emission) vs. FL1 (x axis) channel; (**D**) DAPI‐stained cells observed under a fluorescence microscope (magnification x200), red arrows indicate shrunken nuclei with condensed chromatin, yellow arrows indicate irregular shape of nuclei as morphologic signs of apoptosis; (**E**) ApoStat staining for caspase‐dependent apoptosis, fluorescence intensity from the FL1 channel. Experiments were run in triplicate. The representations are based exemplarily on one experiment. For each staining protocol, the respective control (cells treated with BSA_noMg_) is also shown.

Both **6** and **10** showed the same moderate inhibitory properties on cell proliferation (carboxyfluorescein succinimidyl ester (CFSE) staining, Figure 2A), whereas Fc‐diOH featured stronger inhibition of the proliferation, in line with the literature for all ferrocifen‐type anticancer drugs.^27^ Senescence‐associated (SA) β‐galactosidase staining revealed that **6** induces senescence to a minor extent compared to the transition metal‐containing **10** and ferrocifen (Figure 2B).^39^

Cellular senescence is an important mechanism to prevent tumor cell proliferation and might result in cellular death.^40^ However, the detailed role of senescence in general was found to be way more complicated, because this process not only arrests the cell cycle, but it also stimulates the cells to produce numerous growth factors and other proteins (also called senescence‐associated secretory phenotype, SASP), which can, in turn, provoke again tumor promotion but also tissue repair.^41^ Annexin‐V/PI double staining indicates that apoptosis is not the main mode of action for either **6** or **10** (Figure 2C), which is the case for the reference compounds and for ruthenacarboranes.^8^, ^27^, ^42^

These results could be supported by 4′,6‐diamidino‐2‐phenylindole (DAPI) staining, highlighting shrunken nuclei with condensed chromatin as sign of apoptosis (Figure 2D, red arrows), in the case of **10** to a lesser extent than for Fc‐diOH. However, for **6** a prevalent observation is the presence of irregular‐shaped shrunken nuclei (Figure 2D, yellow arrows) indicating sporadic cells with apoptotic morphology. In concordance with the data above, the activation of caspases was only detected upon treatment of MCF‐7 cells with Fc‐diOH (Figure 2E).

**Figure 3:**
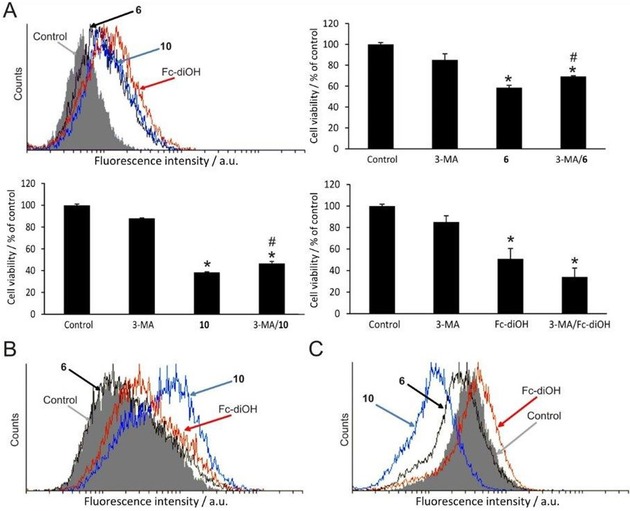
Flow cytometry analysis of MCF‐7 cells exposed (72 h) to an IC_50_ dose of **6**, **10** or Fc‐diOH, respectively, applying the BSA_noMg_ formulation procedure. (**A**) AO staining (upper left panel), fluorescence intensity from the FL3 channel (dark red emission) for detection of acidic vacuoles; cell viability of MCF‐7 cells was determined via CV assays upon treatment with 3‐MA, **6**, **10** or Fc‐diOH and 3‐MA with **6**, **10** or Fc‐diOH. Experiments were run in triplicate. The respective control (cells treated with BSA_noMg_) is also shown. p<0.05 in comparison to * non‐treated cells or # treated with the respective compounds alone (**6**, **10** or Fc‐diOH). (**B**) DAF‐FM (4‐amino‐5‐methylamino‐2′,7′‐difluorofluorescein) staining allows to detect the nitric oxide (NO) level, fluorescence intensity from the FL1 channel; (**C**) DHR 123 staining for determination of the ROS/RNS species, fluorescence intensity from the FL1 channel (green emission). For flow cytometry, experiments were run in triplicate. The representations are based exemplarily on one experiment. For each staining protocol, the respective control (cells treated with BSA_noMg_) is also shown.

Additionally, autophagy processes were triggered by all three tested compounds (Figure 3A); however, different modes of action could be detected after treatment with the autophagy inhibitor 3‐methyladenine (3‐MA). Since Fc‐diOH further lowers the cell viability when applied in combination with 3‐MA, the autophagy process can be considered as cytoprotective (pro‐survival) (Figure 3A, bottom right panel), while the addition of **6** as well as **10** together with 3‐MA resulted in restored viability compared to **6** (or **10**) alone, and it can therefore be regarded as cytodestructive (cell death mediating, Figure 3A, upper right and bottom left panel). This non‐apoptotic and autophagy‐mediated cell death mechanism could be the pathway of how **6** and **10** act on the tested cancer cells. In the literature, also autophagy is discussed as a double‐edged sword, depending on healthy or cancer cell type, microenvironment, and development status.^43^

While in the luminal breast cancer cell line MCF‐7 autophagy seems to be a reasonable cell death process, it was recently shown for other common chemotherapeutics that autophagy in e. g. MDA‐MB‐231 cells is dependent on other signaling pathways.^44^ Autophagy, as one of the main cellular processes, can have a large spectrum of physiological functions affecting cell fate in various ways. Its specific role, however, is determined by the cell specificity, the intensity of triggered processes, as well as the type of the chemotherapeutic that induced it.^45^

To investigate the capability of the drugs to generate reactive oxygen/nitrogen species (ROS/RNS), dihydrorhodamine 123 (DHR 123) staining was performed. It could be shown for Fc‐diOH that the ROS/RNS levels in the treated cells were increased, as it is reported in the literature via the inhibition of cytoprotective radical scavenger thioredoxin,^46^ as well as the self‐generation of ROS/RNS species, which all together lead to apoptosis also in hormone‐independent cell lines.^22^ Similarly, other ferrocene‐containing bioactive molecules induce apoptosis through the generation of ROS.^23^, ^47^ In contrast, both **6** and **10** “scavenge” or lower the cellular ROS/RNS levels (DHR 123, dihydrorhodamine 123 staining; Figure 3C), which corresponds well with the absence of caspase‐dependent apoptosis induction. Worth mentioning is that the molybdacarborane **10** is way more efficient in scavenging/reducing radical concentration compared to its ligand (**6**) only. How exactly **6** and **10** lower the cellular ROS/RNS levels remains unclear and will be addressed in future studies.

Remarkably, compound **10** strongly upregulates the NO production in MCF‐7 cells (Figure 3B). Either it scavenges ROS/RNS and thus generates NO, or it interacts with one or more of the nitric oxide synthases (NOS), as known for other mixed‐sandwich ferracarborane or COSAN‐type structures.^7^ This effect is in high contrast to the other two tested compounds (**6** and Fc‐diOH) in flow cytometry analysis. Also, nitric oxide has an ambivalent role in healthy and cancer cell correlation.^48^ In low concentrations, i. e. 10−300 nm, proliferation of MCF‐7 cells could be induced via HIF‐1α (hypoxia‐induced factor 1α), whereas in higher concentrations, i. e. >300 nm, the p53 tumor protein is further phosphorylated inducing an apoptotic cell death mechanism.^49^

## Conclusions

We could synthesize a 2,2′‐bipyridine vector (**6**) in five synthetic steps that can be employed as ligand for a large variety of organometallic or inorganic transition metal complex fragments giving distinct functions to the whole complex. The activity of the prototypic molybdacarborane [3‐(2,2′‐bipyridine‐κ^2^ *N*,*N*′)‐3‐(CO)_2_‐*closo*‐3,1,2‐MoC_2_B_9_H_11_] (**i**), already active against MCF‐7 breast cancer cell line (IC_50_=ca. 40 μm),^29^ could be improved by incorporating the 2,2′‐bpy‐TAM‐diOH (**6**) ligand. Furthermore, the ligands **3** and **6** were tested in cell viability studies (CV and MTT assays) in comparison to the respective molybdacarborane complexes (**8**–**10**) against breast adenocarcinoma (MDA‐MB‐231, MDA‐MB‐361 and MCF‐7), human glioblastoma (LN‐229) and human glioma (U‐251) cell lines and showed IC_50_ values in low to moderate micromolar range. Importantly, viability of mouse macrophages (Mf) was not significantly affected by the new compounds up to a concentration of 100 μm, in contrast to Fc‐diOH. Modification of the “A” ring in 4,4′‐dihydroxytamoxifen (TAM‐diOH) or ferrocifen (Fc‐diOH) with 2,2′‐bipyridine or [3‐(2,2′‐bipyridine‐κ^2^ *N*,*N*′)‐3‐(CO)_2_‐*closo*‐3,1,2‐MoC_2_B_9_H_11_] modulates the anticancer activity profile significantly. Compounds **6** and **10** were found to have distinctly diverse activities (compared to Fc‐diOH), e. g. induction of senescence, cytodestructive autophagy, lowering of ROS/RNS levels, and, uniquely for **10**, strong increase of nitric oxide (NO) concentration. In perspective, it might be a good chance that the tested compounds are active on different routes rather than causing apoptosis, because most anticancer drugs kill through apoptotic cell death, thus having only little effect on cancer cell lines with defects in the classical apoptotic signaling pathway.^50^ Further details on the mode of action of **6** and **10** are under way.

This study shows that half‐sandwich molybdacarboranes are highly interesting bioactive pharmacophores with distinct activity profile, which can modulate the mode of action of established organic and organometallic anticancer agents. Additionally, it could be shown via Nanoparticle Tracking Analysis (NTA) that the metallacarborane unit is essential for triggering the formation of nanoparticles upon preparation with bovine serum albumin (BSA_noMg_), which might have a positive impact on *in vivo* studies. Thus, both, **6** and **10**, are promising drug candidates for application in breast cancer, glioma and glioblastoma therapy.

## Conflict of interest

The authors declare no conflict of interest.

## Supporting information

As a service to our authors and readers, this journal provides supporting information supplied by the authors. Such materials are peer reviewed and may be re‐organized for online delivery, but are not copy‐edited or typeset. Technical support issues arising from supporting information (other than missing files) should be addressed to the authors.

Supplementary
