# Supplementary material for: 2,2′‐Bipyridine‐Modified Tamoxifen: A Versatile Vector for Molybdacarboranes
Source: ChemMedChem. 2019 Nov 18;14(24):2075–83. doi: 10.1002/cmdc.201900554 (PMC6972990; doi:10.1002/cmdc.201900554)
Supplement: Supplementary file 1 — Supplementary [file CMDC-14-2075-s001.pdf]

CHEMMEDCHEM

Supporting Information

© Copyright Wiley-VCH Verlag GmbH & Co. KGaA, 69451 Weinheim, 2019

2,2′-Bipyridine-Modified Tamoxifen: A Versatile Vector for Molybdacarboranes

Benedikt Schwarze, Sanja Jelača, Linda Welcke, Danijela Maksimović-Ivanić, Sanja Mijatović, and Evamarie Hey-Hawkins*© 2019 The Authors. Published by Wiley-VCH Verlag GmbH & Co. KGaA. This is an open access article under the terms of the Creative Commons Attribution License, which permits use, distribution and reproduction in any medium, provided the original work is properly cited.

Table of Content

1. *Experimental Section* ... *1*
1.1. Methods ... 1
1.2. Instrumentation ... 2
1.3. Syntheses ... 3
2. *X-ray Crystallography* ... *12*
3. *Stability and Solution Behavior Studies* ... *13*
3.1. NMR Spectroscopy ... 13
3.2. UV-Vis, Fluorescence and Resonance Light Scattering (RLS) Spectroscopy ... 19
3.3. Nanoparticle Tracking Analysis (NTA) ... 23
3.4. Mass Spectrometry ... 23
4. *Biological Studies* ... *23*
5. *Cell Viability* ... *26*
6. *References* ... *28*

1. Experimental Section

1.1. Methods

All reactions were carried out under an atmosphere of dry, oxygen-free nitrogen with dry and oxygen-free solvents using Schlenk line techniques. Molecular sieves (3 Å and 4 Å) were activated at 250 °C *in vacuo* for 3 h. Silica gel for inert column chromatography (Merck, 0.035–0.070 mm, 60 Å) was dried (with a heat gun) and degassed *in vacuo* for 6 h. Same silica gel was used for column chromatography in air. Biotage Isolera™ Four was used for flash column chromatography with the corresponding cartridges as indicated for each case. Thin-layer chromatography (TLC) was carried out on precoated glass plates (Merck Silica Gel 60 F254); visualization of the compounds on TLC plates was achieved by means of UV light (254 and 366 nm). Dry, oxygen-free tetrahydrofuran (THF), 1,2-dimethoxyethane (DME) and 1,4-dioxane were distilled from sodium/benzophenone and stored over 4 Å molecular sieves. Dry, oxygen-free acetonitrile (MeCN) and dimethyl sulfoxide (DMSO) were distilled from CaH_2 and stored over 3 Å (MeCN) or over 4 Å (DMSO) molecular sieves. Dry and oxygen-free *N*,*N*-dimethyl formamide (DMF), CH_2Cl_2 (DCM), diethyl ether (Et_2O), *n*-hexane and toluene were obtained from an MBraun Solvent Purification System MB SPS-800, degassed and stored over 3 Å molecular sieves. For NMR measurements, CD_2Cl_2 (DCM-d_2) and $CDCl_3$ were dried by stirring at rt over P_2O_5 for 6 d, degassed by purging with nitrogen for 30 min under ultrasonic conditions and stored over 4 Å molecular sieves. Acetonitrile-d_3 (CD_3CN) was dried by stirring at rt over P_2O_5 for 4 d, followed by vacuum transfer into a storage flask. It was degassed by purging with nitrogen for 30 min applying ultrasonic conditions and stored over 3 Å molecular sieves. The same procedure was applied for DMSO-d_6, but CaH_2 was used instead as drying agent. Chemicals were used as purchased. Dulbecco`s phosphate saline buffer (PBS, 10 mM) was purchased from Biowest (w/o Mg and w/o Ca ions; Product Code: L0615) or freshly prepared in our laboratories according to the technical specification sheet from Dulbecco®. Magnesium- and fatty acids-free bovine serum albumin (BSA_{noMg}) was purchased from SERVA electrophoresis GmbH. The two reference compounds, namely ferrocifen (Fc-diOH)[1] and 1,1-bis(4-hydroxyphenyl)-2-phenylbut-1-ene (TAM-diOH),[2] were synthesized according to the literature. $[NEt_4][3\text{-}(\eta^3\text{-}C_3H_5)\text{-}3\text{-}(CO)_2$-*closo*-3,1,2-$MoC_2B_9H_{11}]$ was synthesized as described elsewhere,[3] as well as 2-(tributylstannyl)pyridine[4] and $[Pd(PPh_3)_4]$.[5]

1.2. Instrumentation

NMR spectra were acquired at room temperature with a Bruker AVANCE III HD 400 spectrometer. ^{1}H (400.13 MHz) and ^{13}C (100.16 MHz) NMR spectra were referenced to tetramethylsilane (TMS) as internal standard. ^{11}B (128.38 MHz) NMR spectra were referenced to the unified Ξ scale.[6] Mass spectrometry measurements were carried out in the ESI-MS mode using a Bruker ESQUIRE 3000 (Benchtop LC Iontrap) spectrometer. IR spectra were obtained with a PerkinElmer system 2000 FTIR spectrometer, scanning between 400 and 4000 cm^{-1} using KBr pellets, which were prepared in a glovebox under nitrogen atmosphere. Elemental analyses were performed with a Hereaus VARIO EL oven. The single crystal X-Ray data were collected on a Gemini-CCD diffractometer (RIGACU INC.) using Mo-K_{α} radiation (λ = 0.71073 Å), ω-scan rotation. Data reduction was performed with CrysAlis Pro[7] including the program SCALE3 ABSPACK[8] for empirical absorption correction. The structure solution for **6** and **9b** was performed with SHELXT (dual-space method).[9] The graphical user interface ShelXle[10] was used for SHELXL.[11] The anisotropic full-matrix least-squares refinement on F^2 of all non-hydrogen atoms was performed with SHELXL-97. Except for disordered solvent molecules, all non-hydrogen atoms were refined with anisotropic thermal parameters and the HFIX command was used to locate all hydrogen atoms for non-disordered regions of the structure. The C_2 unit within the carborane cluster was located with bond lengths analysis. Structure figures were generated with Mercury (version 4.0.0)[12] or UCSF Chimera (version 1.14).[13] CCDC-1944134 (**6**) and CCDC-1944251 (**9b**) contain the supplementary crystallographic data for this paper. These data can be obtained free of charge via https://summary.ccdc.cam.ac.uk/structure-summary-form (or from the Cambridge Crystallographic Data Centre, 12 Union Road, Cambridge CB2 1EZ, UK; fax: (+44)1223-336-033; or deposit@ccdc.cam.uk). UV-Vis absorption spectra were measured with a PerkinElmer UV/VIS/NIR Lambda 900 spectrometer, equipped with tungsten-halogen and deuterium lamps, using quartz cuvettes (V = 3 cm^3, l = 10 mm). Spectra were recorded in the range 240–800 nm, at 1.0 nm resolution. Steady-state fluorescence emission and Rayleigh Light Scattering (RLS) spectra were measured with a PerkinElmer LS-50b spectrometer, equipped with a xenon-arc lamp, using quartz cuvettes (V = 1 cm^3, l = 5 mm). Nanoparticle Tracking Analysis data were recorded using a NanoSight LM10 (Malvern Instruments Ltd, Worcestershire, UK), containing a sample chamber of about 0.25 mL, and equipped with a 532 nm-laser, a microscope LM14B and a camera sCMOS. The NTA 3.0 analytical software (NanoSight Ltd) was used for both capture and processing. Acquisition and processing parameters were optimized for each sample and the respective blank.

1.3. Syntheses

1-(2-Chloropyridin-4-yl)propan-1-one (**2a**)

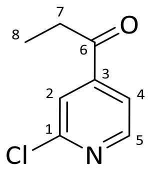

The synthesis was done following Henegar *et al.*[14] In a Schlenk flask 2-chloroisonicotinic acid **1a** (10.0 g, 63.4 mmol, 1.0 equiv.) was suspended in absolute THF (100 mL) and cooled to –40 °C. A solution of ethylmagnesium bromide in Et_2O (3.0 M in Et_2O, 63.5 mL, 190.5 mmol, 3.0 equiv.) was slowly added. The reaction mixture was slowly warmed to 0 °C and stirred at the same temperature for four hours. EtOAc (20 mL) followed by 6.0 M $HCl_{aq.}$ (20 mL) were added to quench the reaction. The phases were separated, and the resulting aqueous phase was extracted with THF (3 x 30 mL). The combined organic phases were subsequently washed with 1.0 M $NaOH_{aq.}$ (2 x 20 mL) and brine (3 x 40 mL) and dried over $MgSO_4$. The solvent was removed under reduced pressure and the crude mixture purified by flash column chromatography on silica with EtOAc/*n*-hexane 1:3 → 1:2 (v/v), to yield **2a** (5.00 g, 29.5 mmol, 47%) as colorless crystals.

R_F (Hex/EtOAc 2:1): 0.55; **^{1}H NMR** (400 MHz, $CDCl_3$): δ (ppm) = 8.57 (d, $^3J_{HH}$ = 5.1 Hz, 1H, 5-C**H**), 7.77 (s, 1H, 2-C**H**), 7.66 (d, $^3J_{HH}$ = 5.0, 1H, 4-C**H**), 2.99 (q, $^3J_{HH}$ = 7.1 Hz, 2H, 7-C**H$_2$**), 1.24 (t, $^3J_{HH}$ = 7.1 Hz, 3H, 8-C**H$_3$**).

1-(2-Bromopyridin-4-yl)propan-1-one (**2b**)

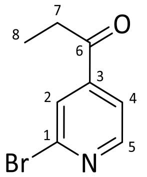

2b was synthesized in an analogous manner as described for **2a**, but using 2-bromoisonicotinic acid **1b** (4.25 g, 20.9 mmol, 1.0 equiv.) and a solution of ethylmagnesium bromide in Et_2O (3.0 M in Et_2O, 25 mL, 62.5 mmol, 3.0 equiv.) in THF (100 mL). The product was purified as described for **2a**, using EtOAc/*n*-hexane 1:3 → 1:2 (v/v), to yield **2b** (1.86 g, 9.00 mmol, 43%) as colorless crystals.

R_F (Hex/EtOAc 2:1): 0.52; **^{1}H NMR** (400 MHz, $CDCl_3$): δ (ppm) = 8.55 (d, $^3J_{HH}$ = 5.0 Hz, 1H, 5-C**H**), 7.92 (s, 1H, 2-C**H**), 7.69 (d, $^3J_{HH}$ = 5.1, 1H, 4-C**H**), 2.98 (q, $^3J_{HH}$ = 7.1 Hz, 2H, 2-C**H$_2$**), 1.24 (t, $^3J_{HH}$ = 7.1 Hz, 3H, 1-C**H$_3$**).

1-[(2,2'-Bipyridin)-4-yl]propan-1-one (**3**)

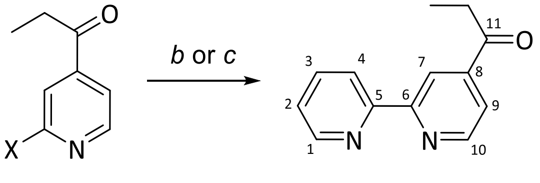

2 **3**

X = Cl (a), Br (b)

Method A (microwave reactions). 1-(2-Chloropyridin-4-yl)propan-1-one (**2a**) (50 mg, 0.29 mmol, 1.0 equiv.) was dissolved in absolute DMF (5 mL) and added to a mixture of [Pd(PPh_3)$_4$] (33.5 mg, 10 mol%), CuI (8.67 mg, 16 mol%) and 2-(tributylstannyl)pyridine (110 µL, 0.34 mmol, 1.2 equiv.). The reaction mixture was placed in a microwave reactor for 16 h at 140 °C. Then CsF (88 mg, 0.58 mmol,

2.0 equiv.) dissolved in distilled water (20 mL) was added to the reaction mixture and stirred for an hour at ambient temperature. The phases were separated, and the aqueous phase was extracted with CH_2Cl_2 (3 x 10 mL). The combined organic phases were washed with H_2O (10 mL) and brine (5 x 20 mL) until the remaining DMF was completely removed from the organic phase. The combined organic phases were dried over $MgSO_4$, filtered and the solvent was removed under reduced pressure. Purification by flash column chromatography on silica gel (25 cm x 2 cm) using an EtOAc/*n*-hexane 1:2 (v/v) with NEt_3 (1%) eluent system yielded compound **3** (42 mg, 0.20 mmol, 69%).

Method B (reflux conditions). Alternatively, 1-(2-chloropyridin-4-yl)propan-1-one (**2a**) (2.46 g, 14.5 mmol, 1.0 equiv.) was dissolved in absolute DMF (ca. 75 mL) together with [Pd(PPh_3)$_4$] (1.68 g, 10 mol%), CuI (0.55 g, 20 mol%), CsF (4.84 g, 31.86 mmol, 2.2 equiv.). Then 2-(tributylstannyl)pyridine (5.1 mL, 15.95 mmol, 1.10 equiv.) was added via syringe and the reaction mixture was kept at 120 °C for 72 h. After cooling to ambient temperature, CH_2Cl_2 (50 mL) and H_2O (50 mL) were added and the mixture filtered into a separation funnel to remove the formed solid. The same workup procedures were applied as for the *microwave reaction* adjusting solvent volumes accordingly. The crude mixture was purified by flash column chromatography using ISOLERA FOUR on silica gel (SNAP Ultra 100g) with an EtOAc/*n*-hexane 8% → 52% EtOAc (v/v) with NEt_3 (1%) eluent system yielding **3** as a colorless solid (1.72 g, 8.10 mmol, 56%).

3 can be also synthesized with the same procedures but using 1-(2-bromopyridin-4-yl)propan-1-one (**2b**) (62.1 mg, 0.29 mmol, 1.0 equiv.) and the other reagents and solvent volumes accordingly.

Table S1. Summary of the optimization process of the STILLE coupling reaction. All reactions were conducted in a total volume of solvent of 5 mL, with 0.29 mmol of 1-(2-X-pyridin-4-yl)propan-1-one (X = Cl, Br) and addition of CuI (16 mol%).

Rct.	X	Catalyst system	CsF	Solvent	MW	T in °C	Reaction time in h	Yield in %
1	Cl	[Pd(PPh_3)$_4$] (2.5 mol%)	–	toluene	Yes	140	19.5	37
2	Cl	[Pd(PPh_3)$_4$] (10 mol%)	–	toluene	Yes	140	18	21
3	Cl	[Pd(PPh_3)$_4$] (10 mol%)	2.0 equiv.	toluene	Yes	140	17	34
4	Cl	[Pd(PPh_3)$_4$] (10 mol%)	2.0 equiv.	DMF	Yes	140	16	69
5	Br	[Pd(PPh_3)$_4$] (10 mol%)	2.0 equiv.	DMF	No	100	19	66
6	Br	[Pd(PPh_3)$_4$] (10 mol%)	2.0 equiv.	DMF	No	140	16	62
7	Br	[Pd(PPh_3)$_4$] (10 mol%)	2.0 equiv.	DMF	No	100	18	65
8	Br	[$PdCl_2$] (10 mol%), [PH(^{t}Bu)$_3$][BF_4] (20 mol%), NEt_3 (20 mol%)	2.0 equiv.	DMF	No	140	16	57

R$_F$ (Hex/EtOAc 2:1 + NEt_3(1%)): 0.35; M($C_{13}H_{12}N_2O$) = 212.25 g mol^{-1}. **Elemental analysis**: $C_{13}H_{12}N_2O$, calc. (%): C 73.56, H 5.70, N 13.20; found (%) C 73.55, H 5.74, N 12.70; **^{1}H NMR** (400 MHz, $CDCl_3$): δ (ppm) = 8.85 (m, 1H, 7-C**H**), 8.83 (d, $^3J_{HH}$ = 5.0 Hz, 1H, 10-C**H**), 8.72 (d, $^3J_{HH}$ = 4.8 Hz, 1H, 1-C**H**), 8.44 (d, $^3J_{HH}$ = 8.1 Hz, 1H, 4-C**H**), 7.85 (ddd, $^3J_{HH}$ = 8.1, $^3J_{HH}$ = 7.7, $^4J_{HH}$ = 1.7 Hz, 1H, 3-C**H**), 7.77 (dd, $^3J_{HH}$ = 5.0, $^4J_{HH}$ = 1.7 Hz, 1H, 9-C**H**), 7.35 (ddd, $^3J_{HH}$ = 7.6, $^3J_{HH}$ = 4.8, $^4J_{HH}$ = 1.2 Hz, 1H, 2-C**H**), 3.13 (q, $^3J_{HH}$ = 7.2 Hz, 2H, 12-C**H$_2$**), 1.27 (t, $^3J_{HH}$ = 7.1 Hz, 3H, 13-C**H$_3$**; **^{13}C{^{1}H} NMR** (101 MHz, $CDCl_3$): δ (ppm) = 200.4 (11-**C**$_{quat.}$), 157.6 (6-**C**$_{quat.}$), 155.4 (5-**C**$_{quat.}$), 150.2 (10-**C**H), 149.3 (1-**C**H), 144.0 (8-**C**$_{quat.}$), 137.1 (3-**C**H), 124.2 (2-**C**H), 121.3 (4-**C**H), 120.8 (9-**C**H), 118.8 (7-**C**H), 32.4 (12-**C**H$_2$), 7.8 (13-**C**H$_3$);); **^{1}H NMR** (400 MHz, DMSO-*d$_6$*): δ (ppm) = 8.90 (d, $^3J_{HH}$ = 4.8 Hz, 1H, 10-C**H**), 8.78 (s, $^3J_{HH}$ = 5.0 Hz, 1H, 7-C**H**), 8.75 (d, $^3J_{HH}$ = 4.8 Hz, 1H, 1-C**H**), 8.42 (d, $^3J_{HH}$ = 8.0 Hz, 1H, 4-C**H**), 7.99 (ddd, $^3J_{HH}$ = 8.0, $^3J_{HH}$ = 7.7, $^4J_{HH}$ = 1.7 Hz, 1H, 3-C**H**), 7.89 (dd, $^3J_{HH}$ = 5.0, $^4J_{HH}$ = 1.7 Hz, 1H, 9-C**H**), 7.51 (ddd, $^3J_{HH}$ = 7.5, $^3J_{HH}$ = 4.8, $^4J_{HH}$ = 1.2 Hz, 1H, 2-C**H**), 3.17 (q, $^3J_{HH}$ = 7.1 Hz, 2H, 12-C**H$_2$**), 1.13 (t, $^3J_{HH}$ = 7.0 Hz, 3H, 13-C**H$_3$**); **IR** (KBr): $\tilde{\nu}$ (cm^{-1}) = 3063 (w, ν(C_{arom}–H)), 3020 (w, ν(C_{arom}–H)), 2974 (w, ν(C_{alkyl}–H)), 2936 (w, ν(C_{alkyl}–H)), 2907 (w, ν(C_{alkyl}–H)), 2013–1806 (vw, arom. overtones), 1699 (s, ν(CO)), 1583 (m, ν(C=C)), 1551 (m, ν(C=C)), 1462 (m, C_{arom}–H in-plane bending), 1412 (m, C_{arom}–H in-plane bending), 1382 (m, C_{arom}–H in-plane bending), 1351 (m, C_{arom}–H in-plane bending),

1284 (w), 1253 (w), 1195 (m), 1093–964 (w), 855 (w), 784 (s, C_{arom}–H out-of-plane bending), 742 (w), 655 (w), 617 (w); **MS** (HR-ESI, pos.): *m/z* = 213.1022 (calc.: 213.1028, $[M+H]^+$).

4-(1,1-Dibromobut-1-en-2yl)-2,2‘-bipyridine (**4a**)

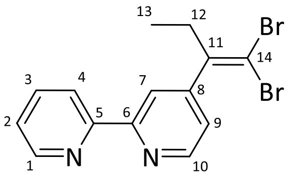

The synthesis was done following Fang *et al.*[15] $P(Oi\text{-}Pr)_3$ (3.5 mL, 14.13 mmol, 3.0 equiv.) was added dropwise with a syringe at 0 °C to a solution of **3** (1.00 g, 4.71 mmol, 1.0 equiv.) and CBr_4 (2.35 g, 7.07 mmol, 1.5 equiv.) dissolved in absolute CH_2Cl_2 (50 mL) (color changed from yellow to red). The mixture was allowed to slowly warm up to ambient temperature and stirred at 40 °C for four days. The yellow to brown reaction mixture was quenched with aq. saturated $NaHCO_3$ solution (50 mL). The two phases were separated, and the aqueous phase was extracted with Et_2O (3 x 30 mL). The combined organic phases were dried over $MgSO_4$, filtered and the solvent was removed under reduced pressure. The crude mixture was purified by flash column chromatography on silica gel (37 cm x 4 cm) with an EtOAc/*n*-hexane (1:3) with NEt_3 (1%) eluent system yielding **4a** as an off-white solid (1.35 g, 3.67 mmol, 78%). Recrystallization from hot *n*-hexane afforded **4a** in high purity as colorless crystals.

$\mathbf{R_F}$ (Hex/EtOAc 2:1 + NEt_3 (1%)): 0.52; M($C_{14}H_{12}Br_2N_2$) = 368.07 g mol^{-1}. **Elemental analysis**: $C_{14}H_{12}Br_2N_2$, calc. (%): C 45.69, H 3.29, N 7.61; found (%) C 46.01, H 3.14, N 7.65; **^{1}H NMR** (400 MHz, $CDCl_3$): δ (ppm) = 8.79 (m, 2H, 10-C**H**, 1-C**H**), 8.44 (d, $^3J_{HH}$ = 7.9 Hz, 1H, 4-C**H**), 8.28 (s, 1H, 7-C**H**), 7.83 (ddd, $^3J_{HH}$ = 8.5, $^3J_{HH}$ = 7.8, $^4J_{HH}$ = 1.8 Hz, 1H, 3-C**H**), 7.32 (ddd, $^3J_{HH}$ = 7.6, $^3J_{HH}$ = 4.8, $^4J_{HH}$ = 1.2 Hz, 1H, 2-C**H**), 7.15 (dd, $^3J_{HH}$ = 5.0, $^4J_{HH}$ = 1.7 Hz, 1H, 9-C**H**), 2.66 (q, $^3J_{HH}$ = 7.5 Hz, 2H, 12-C$\mathbf{H_2}$), 1.02 (t, $^3J_{HH}$ = 7.6 Hz, 3H, 13-C$\mathbf{H_3}$); **^{13}C{^{1}H} NMR** (101 MHz, $CDCl_3$): δ (ppm) = 156.5 (6-$\mathbf{C}_{quat.}$), 155.7 (5-$\mathbf{C}_{quat.}$), 149.9 (8-$\mathbf{C}_{quat.}$), 149.4 (10-**C**H or 1-C**H**), 149.2 (10-**C**H or 1-C**H**), 146.6 (11-$\mathbf{C}_{quat.}$), 137.0 (3-**C**H), 123.9 (2-**C**H), 123.0 (9-**C**H), 121.2 (4-**C**H), 120.1 (7-**C**H), 89.0 (14-$\mathbf{C}_{quat.}$), 32.4 (12-**C**H_2), 11.4 (13-**C**H_3); **IR** (KBr): $\tilde{\nu}$ (cm^{-1}) = 3045 (w, ν(C_{arom}–H)), 3000 (w, ν(C_{arom}–H)), 2972 (w, ν(C_{alkyl}–H)), 2930 (w, ν(C_{alkyl}–H)), 2872 (w, ν(C_{alkyl}–H)), 1981–1728 (vw, arom. overtones), 1600 (m, ν(C=C)), 1582 (s, ν(C=C)), 1564 (m, ν(C=C)), 1541 (m, ν(C=C)), 1456 (s, C_{arom}–H in-plane bending), 1384 (s, C_{arom}–H in-plane bending), 1256 (w), 1105 (w), 1068 (w), 1041 (w), 991 (w), 954 (w), 911 (w), 894 (w), 859 (w), 814 (s, C_{arom}–H out-of-plane bending), 790 (s, C_{arom}–H out-of-plane bending), 738 (s, C_{arom}–H out-of-plane bending), 673 (w), 656 (w), 617 (w), 569 (w); **MS** (HR-ESI, pos.) *m/z* = 368.9434 (calc.: 368.9425; $[M+H]^+$), 390.9210 (calc.: 390.9244; $[M+Na]^+$).

4-(1,1-Dichlorobut-1-en-2yl)-2,2‘-bipyridine (**4b**)

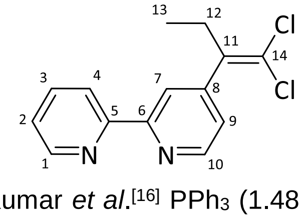

The synthesis was done following Kumar *et al.*[16] PPh_3 (1.48 g, 5.65 mmol, 6.0 equiv.) in absolute toluene (15 mL) CCl_4 (0.28 mL, 2.83 mmol, 3.0 equiv.) was added with a syringe to a solution of **3** (0.20 g, 0.94 mmol, 1.0 equiv.). The reaction mixture was stirred at 110 °C for three days. After cooling to rt the mixture was diluted with CH_2Cl_2 and exposed to air. The solvent was removed under reduced pressure and the crude product was loaded on silica gel to perform column chromatography (ISOLERA ONE) using a gradient of 6% → 50% EtOAc (v/v) with NEt_3 (1%) resulting in **4b** as an off-white solid (0.14 g, 0.64 mmol, 68%). Recrystallization from hot *n*-hexane did not afford complete

separation of the joint product, namely P(O)Ph$_3$, from **4b**. However, the identified impurity does not disturb the next reaction step.

R$_F$ (Hex/EtOAc 3:1 + NEt$_3$ (2%)): 0.66; M($C_{14}H_{12}Cl_2N_2$) = 279.16 g mol^{-1}; **^{1}H NMR** (400 MHz, CDCl$_3$): δ (ppm) = 8.69 (m, 2H, 10-C**H**, 1-C**H**), 8.43 (d, $^3J_{HH}$ = 7.9 Hz, 1H, 4-C**H**), 8.31 (s, 1H, 7-C**H**), 7.82 (ddd, $^3J_{HH}$ = 8.3, $^3J_{HH}$ = 7.6, $^4J_{HH}$ =1.7 Hz, 1H, 3-C**H**), 7.31 (ddd, $^3J_{HH}$ = 7.6, $^3J_{HH}$ = 4.8, $^4J_{HH}$ = 1.2 Hz, 1H, 2-C**H**), 7.17 (dd, $^3J_{HH}$ = 5.0, $^4J_{HH}$ = 1.7 Hz, 1H, 9-C**H**), 2.66 (q, $^3J_{HH}$ = 7.5 Hz, 2H, 12-C**H$_2$**), 1.01 (t, $^3J_{HH}$ = 7.6 Hz, 3H, 13-C**H$_3$**); **MS** (HR-ESI, pos.) *m/z* = 279.045 (calc.: 279.046; [M+H]$^+$).

4-[1,1-Bis(4-methoxyphenyl)but-1-en-2-yl]-2,2'-bipyridine (**5**)

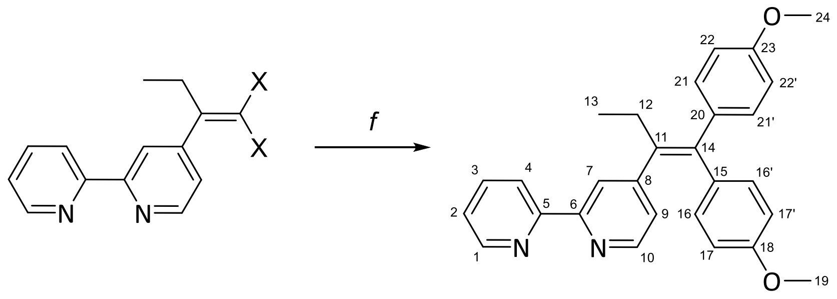

4 **5**

X = Cl (a) , Br (b)

In a 100 mL 2-neck round bottom flask with Schlenk adapter **4a** (0.27 g, 0.73 mmol, 1.0 equiv.), 4-methoxyphenylboronic acid (0.56 g, 3.67 mmol, 5.0 equiv.), Na_2CO_3 (0.39 g, 3.67 mmol, 5.0 equiv.) and [Pd(PPh$_3$)$_4$] (0.09 g, 0.07 mmol, 10 mol%) were dissolved in distilled 1,4-dioxane (16 mL) and degassed, distilled water (4 mL) and stirred at 100 °C for four days. Ethylacetate (EtOAc; 20 mL) and H_2O (20 mL) were added to the orange reaction mixture. The two phases were separated, and the aqueous phase extracted with EtOAc (3 x 20 mL). All combined organic phases were washed with brine (3 x 25 mL) and dried over $MgSO_4$, filtered, and the solvent removed under reduced pressure. The crude mixture was purified by flash column chromatography using ISOLERA FOUR on silica gel (SNAP Ultra 25g) with an EtOAc/*n*-hexane 2% → 20% EtOAc (v/v) with NEt$_3$ (3%) eluent system yielding **5** as colorless solid (0.23 g, 0.54 mmol, 73%). Larger scale: **4a** (1.00 g, 2.71 mmol), yield: **5** (0.75 g, 65%).

Table S2. Summary of the optimization process of the Suzuki coupling reaction. All reactions were conducted in a total solvent volume of 10 mL, with 0.065 mmol of **5** (1.0 equiv.) and 4-methoxyphenylboronic acid (0.26 mmol, 4.0 equiv.).

Rct.	Catalyst system	Base	Solvent	MW	T in °C	Reaction time in h	Yield in %
1	[Pd(PPh$_3$)$_4$] (20 mol%)	CsF	DME	Yes	100	10	-
2	[Pd(dppf)Cl] (12 mol%),[a] BTEAC[b] (2.2 equiv.)	Cs_2CO_3	THF/H_2O (4:1)	No	70	42	65
3	[Pd(dppf)Cl] (12 mol%),[a] BTEAC[b] (2.2 equiv.)	Cs_2CO_3	1,4-dioxane/ H_2O (4:1)	No	100	20	45
4	[Pd(PPh$_3$)$_4$] (12 mol%), BTEAC[b] (2.2 equiv.)	Cs_2CO_3	1,4-dioxane/ H_2O (4:1)	No	100	20	50
5	[Pd(PPh$_3$)$_4$] (10 mol%)	Na_2CO_3	1,4-dioxane /H_2O (4:1)	No	100	96	73

[a] dppf = 1,1'-bis(diphenylphosphino)ferrocene,
[b] BTEAC = benzyltriethylammonium chloride

R_F (Hex/EtOAc 8:1 + 3% NEt_3): 0.30; M($C_{28}H_{26}N_2O_2$) = 422.53 g mol^{-1}; **Elemental analysis**: $C_{28}H_{26}N_2O_2$, calc. (%): C 79.59, H 6.20, N 6.63; found (%) C 79.11, H 6.27, N 6.31; **^{1}H NMR** (400 MHz, $CDCl_3$): δ (ppm) = 8.67 (d, $^3J_{HH}$ = 4.2 Hz, 1H, 1-C**H**), 8.40 (d, $^3J_{HH}$ = 5.0 Hz, 1H, 10-C**H**), 8.35 (d, $^3J_{HH}$ = 8.0 Hz, 1H, 4-C**H**), 8.30 (s, 1H, 7-C**H**), 7.80 (ddd, $^3J_{HH}$ = 8.2, $^3J_{HH}$ = 7.7, $^4J_{HH}$ = 1.7 Hz, 1H, 3-C**H**), 7.29 (ddd, $^3J_{HH}$ = 7.9, $^3J_{HH}$ = 4.8, $^4J_{HH}$ = 1.1 Hz, 1H, 2-C**H**), 7.16 (d, $^3J_{HH}$ = 8.7 Hz, 2H, 21,21'-C**H**), 6.93 (dd, $^3J_{HH}$ = 5.1, $^4J_{HH}$ = 1.7 Hz, 1H, 9-C**H**), 6.89 (d, $^3J_{HH}$ = 8.6 Hz, 2H, 22,22'-C**H**), 6.84 (d, $^3J_{HH}$ = 8.7 Hz, 2H, 16,16'-C**H**), 6.56 (d, $^3J_{HH}$ = 8.7 Hz, 2H, 17,17'-C**H**), 3.83 (s, 3H, 24-C**H₃**), 3.66 (s, 3H, 19-C**H₃**), 2.59 (q, $^3J_{HH}$ = 7.5 Hz, 2H, 12-C**H₂**), 0.97 (t, $^3J_{HH}$ = 7.4 Hz, 3H, 13-C**H₃**); **^{13}C{^{1}H} NMR** (101 MHz, $CDCl_3$) δ (ppm) = 158.6 (23-**C**$_{quat.}$), 158.0 (18-**C**$_{quat.}$), 156.3 (5-**C**$_{quat.}$), 155.9 (6-**C**$_{quat.}$), 152.3 (8-**C**$_{quat.}$), 149.1 (1-**C**H), 148.7 (10-**C**H), 140.2 (14-**C**$_{quat.}$), 138.7 (11-**C**$_{quat.}$), 136.9 (3-**C**H), 135.5 (20-**C**$_{quat.}$), 134.8 (15-**C**$_{quat.}$), 132.0 (16,16'-**C**H), 130.5 (21,21'-**C**H), 125.8 (9-**C**H), 123.6 (2-**C**H), 121.3 (7-**C**H), 121.1 (4-**C**H), 113.6 (22,22'-**C**H), 113.1 (17,17'-**C**H), 55.2 (24-**C**H₃), 55.0 (19-**C**H₃), 28.4 (12-**C**H₂), 13.6 (13-**C**H₃); **^{1}H NMR** (400 MHz, DMSO-*d₆*): δ (ppm) = 8.64 (d, $^3J_{HH}$ = 4.5 Hz, 1H, 1-C**H**), 8.47 (d, $^3J_{HH}$ = 5.0 Hz, 1H, 10-C**H**), 8.34 (d, $^3J_{HH}$ = 8.0 Hz, 1H, 4-C**H**), 8.18 (s, 1H, 7-C**H**), 7.92 (ddd, $^3J_{HH}$ = 8.0, $^3J_{HH}$ = 7.7, $^4J_{HH}$ = 1.6 Hz, 1H, 3-C**H**), 7.43 (dd, $^3J_{HH}$ = 7.6, $^3J_{HH}$ = 4.8 Hz, 1H, 2-C**H**), 7.16 (m, 1H, 9-C**H**), 7.15 (d, $^3J_{HH}$ = 8.7 Hz, 2H, 21,21'-C**H**), 6.97 (d, $^3J_{HH}$ = 8.7 Hz, 2H, 22,22'-C**H**), 6.81 (d, $^3J_{HH}$ = 8.7 Hz, 2H, 16,16'-C**H**), 6.63 (d, $^3J_{HH}$ = 8.8 Hz, 2H, 17,17'-C**H**), 3.78 (s, 3H, 24-C**H₃**), 3.59 (s, 3H, 19-C**H₃**), 2.48 (q, $^3J_{HH}$ = 7.4 Hz, 2H, 12-C**H₂**), 0.90 (t, $^3J_{HH}$ = 7.4 Hz, 3H, 13-C**H₃**); **^{13}C{^{1}H} NMR** (101 MHz, DMSO-*d₆*) δ (ppm) = 158.1 (23-**C**$_{quat.}$), 157.5 (18-**C**$_{quat.}$), 155.0 (5-**C**$_{quat.}$), 154.9 (6-**C**$_{quat.}$), 151.5 (8-**C**$_{quat.}$), 149.1 (1-**C**H), 148.8 (10-**C**H), 139.6 (14-**C**$_{quat.}$), 138.0 (11-**C**$_{quat.}$), 137.1 (3-**C**H), 134.7 (20-**C**$_{quat.}$), 134.3 (15-**C**$_{quat.}$), 131.3 (16,16'-**C**H), 130.0 (21,21'-**C**H), 125.1 (9-**C**H), 124.1 (2-**C**H), 120.7 (7-**C**H), 120.3 (4-**C**H), 113.6 (22,22'-**C**H), 113.1 (17,17'-**C**H), 55.0 (24-**C**H₃), 54.7 (19-**C**H₃), 28.0 (12-**C**H₂), 13.1 (13-**C**H₃); **IR** (KBr): $\tilde{\nu}$ (cm^{-1}) = 3049 (w, ν(C_{arom}–H)), 3006 (w, ν(C_{arom}–H)), 2954 (w, ν(C_{alkyl}–H)), 2930 (w, ν(C_{alkyl}–H)), 2868 (w, ν(C_{alkyl}–H)), 2838 (w, ν(C_{alkyl}–H)), 2042–1889 (vw, arom. overtones), 1607 (m, ν(C=C)), 1580 (m, ν(C=C)), 1538 (w, ν(C=C)), 1508 (s, C_{arom}–H in-plane bending), 1456 (m, C_{arom}–H in-plane bending), 1389 (w, C_{arom}–H in-plane bending), 1297 (w), 1271 (w), 1244 (s, C_{arom}–O), 1171 (m, $C_{arom.}$–O), 1106 (w), 1032 (m, C_{alkyl}–O), 828 (m, C_{arom}–H out-of-plane bending), 793 (m, C_{arom}–H out-of-plane bending), 739 (w), 617 (w), 589 (w), 570 (w), 519 (w); **MS** (HR-ESI, pos.) *m/z* = 423.2070 (calc.: 423.2073; [M+H]$^+$).

4-[1,1-Bis(4-hydroxyphenyl)but-1-en-2-yl]-2,2'-bipyridine (**6**)

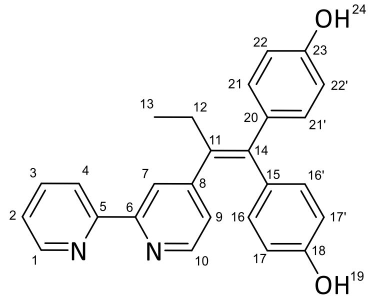

In a Schlenk flask **5** (0.180 g, 0.456 mmol, 1.0 equiv.) was dissolved in CH_2Cl_2 (10 mL) and cooled to –65 °C. BBr_3 (0.26 mL, 2.74 mmol, 6.0 equiv.) was added slowly to the mixture via syringe and the mixture was slowly warmed up to ambient temperature and then stirred for 12 h (the color of the reaction mixture changed from orange/red to light orange and the formation of a precipitate was observed). The reaction was quenched with H_2O (20 mL), the two phases were separated, and the aqueous phase was extracted with EtOAc (3 x 20 mL). All combined organic phases were dried over $MgSO_4$, filtered and the solvent was removed under reduced pressure. Flash column chromatography on silica gel (22 cm x 3 cm) using a $CHCl_3$/MeOH/$NH_{3(aq.)}$ 8:1:0.1 (v/v) eluent system yielded a pale-yellow sticky solid, which was recrystallized from hot $CHCl_3$ with a few drops of MeOH to give pale yellow crystals of **6** (0.143 g, 80%).

R$_F$ ($CHCl_3$/MeOH/$NH_{3(aq.)}$ 8:1:0.1): 0.5; M($C_{26}H_{22}N_2O_2$) = 394.17 g mol^{-1}; **Elemental analysis**: $C_{26}H_{22}N_2O_2 \cdot H_2O$, calc. (%): C 75.71, H 5.86, N 6.79; found (%) C 75.84, H 5.53, N 6.63; **^{1}H NMR** (400 MHz, CD_3OD): δ (ppm) = 8.61 (d, $^3J_{HH}$ = 4.4 Hz, 1H, 1-C**H**), 8.37 (d, $^3J_{HH}$ = 5.1 Hz, 1H, 10-C**H**), 8.16 (d, $^3J_{HH}$ = 8.0 Hz, 1H, 4-C**H**), 8.10 (s, 1H, 7-C**H**), 7.90 (ddd, $^3J_{HH}$ = 8.1, $^3J_{HH}$ = 7.8, $^4J_{HH}$ = 1.6 Hz, 1H, 3-C**H**), 7.41 (dd, $^3J_{HH}$ = 7.7, $^4J_{HH}$ = 4.6 Hz, 1H, 2-C**H**), 7.11 (dd, $^3J_{HH}$ = 5.1, $^4J_{HH}$ = 1.4 Hz, 1H, 9-C**H**), 7.06 (d, $^3J_{HH}$ = 8.5 Hz, 2H, 21,21'-C**H**), 6.79 (d, $^3J_{HH}$ = 8.5 Hz, 2H, 22,22'-C**H**), 6.73 (d, $^3J_{HH}$ = 8.6 Hz, 2H, 16,16'-C**H**), 6.46 (d, $^3J_{HH}$ = 8.6 Hz, 2H, 17,17'-C**H**), 2.61 (q, $^3J_{HH}$ = 7.4 Hz, 2H, 12-C**H$_2$**), 0.97 (t, $^3J_{HH}$ = 7.4 Hz, 3H, 13-C**H$_3$**); **^{13}C{^{1}H} NMR** (101 MHz, methanol-*d$_4$*) δ (ppm) = 157.9 (23-**C**$_{quat.}$), 157.3 (18-**C**$_{quat.}$), 157.2 (5-**C**$_{quat.}$), 156.8 (6-**C**$_{quat.}$), 154.9 (8-**C**$_{quat.}$), 150.2 (1-**C**H), 149.7 (10-**C**H), 143.2 (14-**C**$_{quat.}$), 138.8 (11-**C**$_{quat.}$), 138.7 (3-**C**H), 135.5 (20-**C**$_{quat.}$), 135.2 (15-**C**$_{quat.}$), 133.2 (16,16'-**C**H), 131.6 (21,21'-**C**H), 127.1 (9-**C**H), 125.2 (2-**C**H), 123.8 (7-**C**H), 122.7 (4-**C**H), 115.9 (22,22'-**C**H), 115.5 (17,17'-**C**H), 29.1 (12-**C**H$_2$), 14.0 (13-**C**H$_3$); **^{1}H NMR** (400 MHz, DMSO-*d$_6$*): δ (ppm) = 9.48 (s, 1H, 19-O**H**), 9.22 (s, 1H, 24-O**H**), 8.63 (d, $^3J_{HH}$ = 4.8 Hz, 1H, 1-C**H**), 8.45 (d, $^3J_{HH}$ = 5.0 Hz, 1H, 10-C**H**), 8.34 (d, $^3J_{HH}$ = 8.0 Hz, 1H, 4-C**H**), 8.14 (s, 1H, 7-C**H**), 7.91 (ddd, $^3J_{HH}$ = 8.1, $^3J_{HH}$ = 7.9, $^4J_{HH}$ = 1.8 Hz, 1H, 3-C**H**), 7.42 (dd, $^3J_{HH}$ = 7.7, $^4J_{HH}$ = 4.6 Hz, 1H, 2-C**H**), 7.12 (dd, $^3J_{HH}$ = 5.1, J_{HH} = 1.6 Hz, 1H, 9-C**H**), 7.02 (d, $^3J_{HH}$ = 8.5 Hz, 2H, 21,21'-C**H**), 6.77 (d, $^3J_{HH}$ = 8.5 Hz, 2H, 22,22'-C**H**), 6.67 (d, $^3J_{HH}$ = 8.6 Hz, 2H, 16,16'-C**H**), 6.43 (d, $^3J_{HH}$ = 8.6 Hz, 2H, 17,17'-C**H**), 2.49 (q, $^3J_{HH}$ = 7.4 Hz, 2H, 12-C**H$_2$**), 0.88 (t, $^3J_{HH}$ = 7.4 Hz, 2H, 13-C**H$_3$**); **IR** (KBr): ṽ (cm^{-1}) = 3410 (m, ν(O–H)), 3063 (m, ν(C_{arom}–H)), 2964 (m, ν(C_{alkyl}–H)), 2928 (m, ν(C_{alkyl}–H)), 2869 (m, ν(C_{alkyl}–H)), 2784 (m, ν(C_{alkyl}–H)), 2717 (m, ν(C_{alkyl}–H)), 2662 (m, ν(C_{alkyl}–H)), 2593 (m, ν(C_{alkyl}–H)), 2100–1889 (vw, arom. overtones), 1608 (s, ν(C=C)), 1587 (s, ν(C=C)), 1538 (w ν(C=C)), 1511 (s, C_{arom}–H in-plane bending), 1464 (m, C_{arom}–H in-plane bending), 1439 (m, C_{arom}–H in-plane bending), 1389 (m, C_{arom}–H in-plane bending), 1374 (m, C_{arom}–H in-plane bending), 1270 (s, C_{arom}–O), 1238 (s, C_{arom}–O), 1171 (m, C_{arom}–O), 1102 (w), 1071 (w), 1002 (w), 833 (m, C_{arom}–H out-of-plane bending), 792 (m, C_{arom}–H out-of-plane bending), 753 (m), 591 (w); **MS** (HR-ESI, pos.) *m/z* = 395.1730 (calc.: 395.1760; [M+H]$^+$), 417.1628 (calc.: 417.1579; [M+Na]$^+$), 811.3373 (calc.: 811.3260; [2M+Na]$^+$); (neg.) *m/z* = 393.1598 (calc.: 393.1603; [M–H]$^-$).

[3,3-{1-[(2,2'-Bipyridin-κ^2*N*,*N'*)-4-yl]propan-1-one}-3-(CO)$_2$-*closo*-3,1,2-MoC$_2$B$_9$H$_{11}$] (**8**)

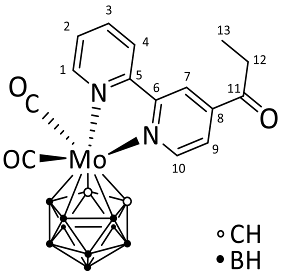

A stock solution of [NEt_4][3-(η^3-C_3H_5)-3-(CO)$_2$-*closo*-3,1,2-Mo$C_2B_9H_{11}$] (**7**) (7.5 mL, 1.49 mmol, 1.0 equiv.) in THF (0.2 M) was treated with HCl in Et_2O (2 M in Et_2O, 1.1 mL, 2.24 mmol, 1.5 equiv.) at 0 °C and the mixture was stirred for 30 min at the same temperature, and then for 30 min at rt. **4** (0.317 g, 1.49 mmol, 1.0 equiv.) dissolved in THF (5 mL) was then added to the green reaction mixture and then stirred at 50 °C for 16 h (color change to purple). The solvent was removed under reduced pressure and the residue dried in vacuum for 1 h. The crude residue was exposed to air and purified by flash column chromatography (applying N_2 gas) on silica gel (29 cm x 4 cm) using pure CH_2Cl_2 as eluent. The third violet band (R$_F$ (CH_2Cl_2) = 0.6) was collected yielding **8** as a dark violet solid (0.136 g, 18%).

R$_F$ (CH_2Cl_2): 0.6; M($C_{17}H_{23}B_9MoN_2O_3$) = 496.63 g mol^{-1}; **Elemental analysis**: $C_{17}H_{23}B_9MoN_2O_3$, calc. (%): C 41.11, H 4.67, N 5.64; found (%) C 40.94, H 4.74, N 5.37; **^{1}H NMR** (400 MHz, CD_2Cl_2): δ (ppm) = 9.28 (d, $^3J_{HH}$ = 6.0 Hz, 1H, 10-C**H**), 9.14 (d, $^3J_{HH}$ = 5.9 Hz, 1H, 1-C**H**), 8.78 (s, 1H, 7-C**H**), 8.53 (d, $^3J_{HH}$ = 8.2 Hz, 1H, 4-C**H**), 8.12 (dd, $^3J_{HH}$ = 8.3, $^3J_{HH}$ = 7.7 Hz, 1H, 3-C**H**), 7.85 (d, $^3J_{HH}$ = 6.1 Hz, 1H, 9-C**H**), 7.56 (dd, $^3J_{HH}$ = 7.9, $^3J_{HH}$ = 6.0 Hz, 1H, 2-C**H**), 3.12 (q, $^3J_{HH}$ = 7.0 Hz, 2H, 12-C**H$_2$**), 2.48 (br s, 1H,

C**H**$_{cluster}$), 2.46 (br s, 1H, C**H**$_{cluster}$), 1.27 (t, $^3J_{HH}$ = 7.0 Hz, 3H, 13-C**H$_3$**), 0.66–3.71 (m, 9H, B**H**$_{cluster}$); **^{13}C{^{1}H} NMR** (101 MHz, CD_2Cl_2): δ (ppm) = 255.3 (**C**O), 254.5 (**C**O), 198.0 (11-**C**$_{quat.}$), 156.8 (10-**C**H), 155.9 (1-**C**H), 153.0 (6-**C**$_{quat.}$), 152.4 (5-**C**$_{quat.}$), 143.7 (8-**C**$_{quat.}$), 138.5 (3-**C**H), 125.9 (2-**C**H), 124.3 (4-**C**H), 122.4 (9-**C**H), 121.1 (7-**C**H), 54.8 (br, **C**$_{cluster}$–H), 33.0 (12-**C**H$_2$), 7.8 (13-**C**H$_3$); **^{11}B NMR** (128 MHz, CD_2Cl_2): δ (ppm) = 7.6 (d, $^1J_{BH}$ = 120 Hz, 1B, **B**H), –2.4 (d, $^1J_{BH}$ = 131 Hz, 2B, **B**H), –5.1 (d, $^1J_{BH}$ = 138 Hz, 1B, B–H), –12.2 (d, $^1J_{BH}$ = 128 Hz, 4B, **B**H), –17.2 (d, $^1J_{BH}$ = 130 Hz, 1B, **B**H); **^{1}H NMR** (400 MHz, DMSO-*d$_6$*): δ (ppm) = 9.12 (m, 3H, 4-C**H**, 7-C**H**, 10-C**H**), 9.00 (d, $^3J_{HH}$ = 5.9 Hz, 1H, 1-C**H**), 8.30 (dd, $^3J_{HH}$ = 8.3, $^3J_{HH}$ = 7.5 Hz, 1H, 3-C**H**), 8.02 (d, $^3J_{HH}$ = 6.1 Hz, 1H, 9-C**H**), 7.74 (dd, $^3J_{HH}$ = 7.9, $^3J_{HH}$ = 6.0 Hz, 1H, 2-C**H**), 3.25 (q, $^3J_{HH}$ = 7.0 Hz, 2H, 12-C**H$_2$**), 2.94 (br s, 1H, C**H**$_{cluster}$), 2.88 (br s, 1H, C**H**$_{cluster}$), 1.15 (t, $^3J_{HH}$ = 7.1 Hz, 3H, 13-C**H$_3$**), 0.66–3.71 (m, 9H, B**H**$_{cluster}$); **IR** (KBr): $\tilde{\nu}$ (cm^{-1}) = 3050–3020 (vw, ν(C$_{arom}$–H)), 2974–2850 (vw, ν(C$_{alkyl}$–H)), 2595–2480 (m, ν(B–H)), 1954 (s, ν(CO)), 1886 (s, ν(CO)), 1700 (m, ν(CO)), 1635 (w, ν(C=C)), 1607 (m, ν(C=C)), 1418–1257 (w, C$_{arom}$–H in-plane bending), 1199 (m), 1149–883 (w), 785 (s, C$_{arom}$–H out-of-plane bending or ν(B–B)), 745 (w); **MS** (HR-ESI, pos.) *m/z* = 442.1827 (calc.: 442.1889; [M–2CO+H]$^+$); 515.1991 (calc.: 515.2053; [M+NH$_4$]$^+$); 1012.3647 (calc.: 1012.3658; [2M+NH$_4$]$^+$).

[3,3-{4-[1,1-Bis(4-methoxyphenyl)but-1-en-2-yl]-2,2'-bipyridine-κ^2*N*,*N'*}-3-(CO)$_2$-*closo*-3,1,2-MoC$_2$B$_9$H$_{11}$] (9)

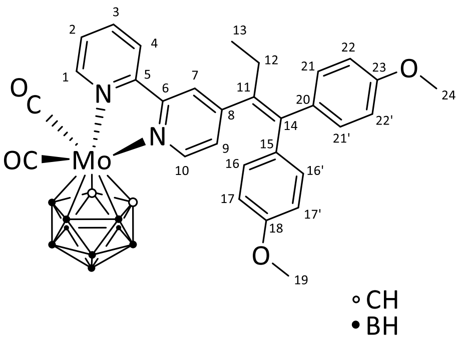

The same procedure was applied as for **8**, but using [NEt$_4$][3-(η^3-C$_3$H$_5$)-3-(CO)$_2$-*closo*-3,1,2-MoC$_2$B$_9$H$_{11}$] (**7**) (5.0 mL, 1.00 mmol, 1.0 equiv.) in THF (0.2 M), HCl in Et_2O (2 M in Et_2O, 0.75 mL, 1.50 mmol, 1.5 equiv.) and **5** (0.423 g, 1.00 mmol, 1.0 equiv.). Flash column chromatography (applying N_2 gas) was performed on silica gel (36 cm x 4 cm) using CH_2Cl_2/*n*-hexane (8:1) → CH_2Cl_2. The first violet band was collected (R_F (CH_2Cl_2): 0.8) yielding **9** as a dark violet solid (0.140 g, 20%). Additionally, the second violet band (R_F (CH_2Cl_2): 0.67) was collected and could be identified as the B(8)-substituted chloro derivative (**9b**).

R$_F$ (CH_2Cl_2): 0.8; M($C_{32}H_{37}B_9MoN_2O_4$) = 706.91 g mol^{-1}; **Elemental analysis**: $C_{32}H_{37}B_9MoN_2O_4$, calc. (%): C 54.37, H 5.28, N 3.96; found (%) C 55.17, H 5.42, N 3.57; **^{1}H NMR** (400 MHz, $CDCl_3$): δ (ppm) = 9.10 (d, $^3J_{HH}$ = 5.5 Hz, 1H, 1-C**H**), 8.79 (d, $^3J_{HH}$ = 5.9 Hz, 1H, 10-C**H**), 8.02 (m, 1H, 4-C**H**), 8.01 (m, 1H, 7-C**H**), 7.93 (dd, $^3J_{HH}$ = 8.8, $^3J_{HH}$ = 7.4 Hz, 1H, 3-C**H**), 7.43 (dd, $^3J_{HH}$ = 7.7, $^3J_{HH}$ = 5.7 Hz, 1H, 2-C**H**), 7.17 (m, 1H, 9-C**H**), 7.16 (d, $^3J_{HH}$ = 8.6 Hz, 2H, 21,21'-C**H**), 6.92 (d, $^3J_{HH}$ = 8.7 Hz, 2H, 22,22'-C**H**), 6.82 (d, $^3J_{HH}$ = 8.7 Hz, 2H, 16,16'-C**H**), 6.63 (d, $^3J_{HH}$ = 8.6 Hz, 2H, 17,17'-C**H**), 3.85 (s, 3H, 24-C**H$_3$**), 3.70 (s, 3H, 19-C**H$_3$**), 2.67 (q, $^3J_{HH}$ = 7.5 Hz, 2H, 12-C**H$_2$**), 2.35 (br s, 1H, C**H**$_{cluster}$), 2.26 (br s, 1H, C**H**$_{cluster}$), 1.04 (t, $^3J_{HH}$ = 7.4 Hz, 3H, 13-C**H$_3$**), 0.70–3.90 (m, 9H, B**H**$_{cluster}$); **^{13}C{^{1}H} NMR** (101 MHz, $CDCl_3$) δ (ppm) = 256.3 (**C**O), 255.5 (**C**O), 159.4 (23-**C**$_{quat.}$), 159.2 (18-**C**$_{quat.}$), 155.6 (1-**C**H), 154.6 (10-**C**H), 154.1 (8-**C**$_{quat.}$), 152.4 (5-**C**$_{quat.}$), 151.4 (6-**C**$_{quat.}$), 144.7 (14-**C**$_{quat.}$), 137.7 (3-**C**H), 136.1 (11-**C**$_{quat.}$), 134.5 (20-**C**$_{quat.}$), 134.0 (15-**C**$_{quat.}$), 132.4 (16,16'-**C**H), 130.7 (21,21'-**C**H), 126.6 (9-**C**H), 125.0 (2-**C**H), 124.4 (7-**C**H), 123.0 (4-**C**H), 113.9 (22,22'-**C**H), 113.8 (17,17'-**C**H), 55.5 (24-**C**H$_3$), 55.4 (19-**C**H$_3$), 54.1 (**C**H$_{cluster}$), 53.9 (**C**H$_{cluster}$), 28.1 (12-**C**H$_2$), 14.1 (13-**C**H$_3$); **^{11}B NMR** (128 MHz, $CDCl_3$): δ (ppm) = 7.9 (d, $^1J_{BH}$ = 100 Hz, 1B, **B**H), –2.2 (d, $^1J_{BH}$ = 100 Hz, 2B, **B**H), –5.2 (d, $^1J_{BH}$ = 120 Hz, 1B, **B**H), –12.2 (m, 4B, **B**H), –16.8 (m, 1B, **B**H); **^{1}H NMR** (400 MHz, DMSO-*d$_6$*): δ (ppm) = 8.93 (d, $^3J_{HH}$ = 5.9 Hz, 1H, 1-

C**H**), 8.87 (d, $^3J_{HH}$ = 7.8 Hz, 1H, 10-C**H**), 8.79 (s, 1H, 7-C**H**), 8.57 (d, $^3J_{HH}$ = 6.0 Hz, 1H, 4-C**H**), 8.24 (dd, $^3J_{HH}$ = 8.6, $^3J_{HH}$ = 7.3 Hz, 1H, 3-C**H**), 7.70 (dd, $^3J_{HH}$ = 7.7, $^3J_{HH}$ = 6.0 Hz, 1H, 2-C**H**), 7.16 (d, $^3J_{HH}$ = 8.7 Hz, 2H, 21,21'-C**H**), 7.13 (d, $^3J_{HH}$ = 6.1, $^3J_{HH}$ = 2.0 Hz, 1H, 9-C**H**), 6.99 (d, $^3J_{HH}$ = 8.8 Hz, 2H, 22,22'-C**H**), 6.77 (d, $^3J_{HH}$ = 8.8 Hz, 2H, 16,16'-C**H**), 6.63 (d, $^3J_{HH}$ = 8.7 Hz, 2H, 17,17'-C**H**), 3.79 (s, 3H, 24-C**H$_3$**), 3.64 (s, 3H, 19-C**H$_3$**), 2.91 (br s, 1H, C**H**$_{cluster}$), 2.70 (br s, 1H, C**H**$_{cluster}$), 2.67 (q, $^3J_{HH}$ = 7.5 Hz, 2H, 12-C**H$_2$**), 0.96 (t, $^3J_{HH}$ = 7.3 Hz, 3H, 13-C**H$_3$**), 0.70–3.90 (m, 9H, B**H**$_{cluster}$); **IR** (KBr): $\tilde{\nu}$ (cm^{-1}) = 2957–2836 (w, , ν(C$_{alkyl}$–H)), 2526 (m, ν(B–H)), 1955 (s, ν(CO)), 1874 (s, ν(CO)), 1604 (m, ν(C=C)), 1507 (m, C$_{arom}$–H in-plane bending), 1462 (w, C$_{arom}$–H in-plane bending), 1437 (w, C$_{arom}$–H in-plane bending), 1413 (w, C$_{arom}$–H in-plane bending), 1299 (w), 1280 (w), 1246 (m, C$_{arom}$–O), 1173 (m, C$_{arom}$–O), 1030 (w, C$_{alkyl}$–O), 980 (w), 830 (w, C$_{arom}$–H out-of-plane bending), 786 (w, C$_{arom}$–H out-of-plane bending or ν(B–B), 745 (w), 591 (w), 572 (w), 530 (w), 470 (w); **MS** (HR-ESI, neg.) *m/z* = 742.2409 (calc.: 742.2443 [M+Cl]$^-$), 752.2696 (calc.: 752. 2703 [M+HCOOH–H]$^-$).

[3,3-{4-[1,1-Bis(4-methoxyphenyl)but-1-en-2-yl]-2,2'-bipyridine-κ^2*N*,*N'*}-3-(CO)$_2$-8-chloro-*closo*-3,1,2-MoC$_2$B$_9$H$_{11}$] (9b)

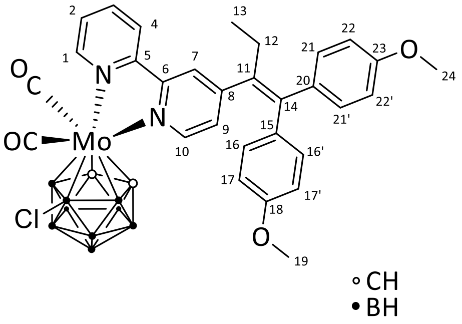

R$_F$ (CH$_2$Cl$_2$): 0.67; M(C$_{32}$H$_{36}$B$_9$ClMoN$_2$O$_4$) = 741.35 g mol^{-1}; **^{1}H NMR** (400 MHz, CDCl$_3$): δ (ppm) = 9.22 (d, $^3J_{HH}$ = 5.8 Hz, 1H, 1-C**H**), 8.91 (d, $^3J_{HH}$ = 6.0 Hz, 1H, 10-C**H**), 8.00 (m, 1H, 4-C**H**), 8.00 (m, 1H, 7-C**H**), 7.97 (m, 1H, 3-C**H**), 7.51 (dd, $^3J_{HH}$ = 7.0, $^3J_{HH}$ = 5.6 Hz, 1H, 2-C**H**), 7.24 (d, $^3J_{HH}$ = 6.1 Hz, 1H, 9-C**H**), 7.16 (d, $^3J_{HH}$ = 8.6 Hz, 2H, 21,21'-C**H**), 6.92 (d, $^3J_{HH}$ = 8.7 Hz, 2H, 22,22'-C**H**), 6.82 (d, $^3J_{HH}$ = 8.7 Hz, 2H, 16,16'-C**H**), 6.63 (d, $^3J_{HH}$ = 8.6 Hz, 2H, 17,17'-C**H**), 3.85 (s, 3H, 24-C**H$_3$**), 3.70 (s, 3H, 19-C**H$_3$**), 2.68 (q, $^3J_{HH}$ = 7.4 Hz, 2H, 12-C**H$_2$**), 2.19 (br s, 1H, C**H**$_{cluster}$), 2.08 (br s, 1H, C**H**$_{cluster}$), 1.04 (t, $^3J_{HH}$ = 7.4 Hz, 3H, 13-C**H$_3$**), 0.70–3.90 (m, 9H, B**H**$_{cluster}$); **^{1}H NMR** (400 MHz, CD$_3$CN) δ (ppm) = 9.14 (d, $^3J_{HH}$ = 5.5 Hz, 1H, 1-C**H**), 8.81 (d, $^3J_{HH}$ = 5.9 Hz, 1H, 10-C**H**), 8.30 (d, $^3J_{HH}$ = 8.1 Hz, 1H, 4-C**H**), 8.27 (s, 1H, 7-C**H**), 8.08 (m, 1H, 3-C**H**), 7.55 (m, 1H, 2-C**H**), 7.21 (m, 1H, 9-C**H**), 7.20 (d, $^3J_{HH}$ = 8.6 Hz, 2H, 21,21'-C**H**), 6.96 (d, $^3J_{HH}$ = 8.4 Hz, 2H, 22,22'-C**H**), 6.84 (d, $^3J_{HH}$ = 8.5 Hz, 2H, 16,16'-C**H**), 6.61 (d, $^3J_{HH}$ = 8.2 Hz, 2H, 17,17'-C**H$_3$**), 3.81 (s, 3H, 24-C**H$_3$**), 3.64 (s, 3H, 19-C**H$_3$**), 2.63 (q, $^3J_{HH}$ = 7.4 Hz, 2H, 12-C**H$_2$**), 2.58 (br s, 1H, C**H**$_{cluster}$), 2.39 (br s, 1H, C**H**$_{cluster}$), 0.98 (t, $^3J_{HH}$ = 7.2 Hz, 3H, 13-C**H$_3$**), 0.75–3.40 (m, 9H, B**H**$_{cluster}$); **^{13}C{^{1}H} NMR** (101 MHz, CD$_3$CN) δ (ppm) = 257.8 (**C**O), 257.4 (**C**O), 157.4 (23-**C**$_{quat.}$), 157.0 (18-**C**$_{quat.}$), 156.5 (1-**C**H), 155.8 (6-**C**$_{quat.}$), 155.3 (10-**C**H), 154.6 (5-**C**$_{quat.}$), 153.8 (8-**C**$_{quat.}$), 160.1 (23-**C**$_{quat.}$), 159.7 (18-**C**$_{quat.}$), 156.5 (1-**C**H), 156.0 (8-**C**$_{quat.}$), 155.3 (10-**C**H), 154.9 (5-**C**$_{quat.}$), 154.2 (6-**C**$_{quat.}$), 144.0 (14-**C**$_{quat.}$), 140.0 (3-**C**H), 138.0 (11-**C**$_{quat.}$), 135.6 (20-**C**$_{quat.}$), 135.1 (15-**C**$_{quat.}$), 133.1 (16,16'-**C**H), 131.3 (21,21'-**C**H), 128.5 (9-**C**H), 126.8 (2-**C**H), 125.8 (7-**C**H), 124.7 (4-**C**H), 114.6 (22,22'-**C**H), 114.2 (17,17'-**C**H), 56.0 (24-**C**H$_3$), 55.8 (19-**C**H$_3$), 53.0 (**C**H$_{cluster}$), 28.6 (12-**C**H$_2$), 13.7 (13-**C**H$_3$); **^{11}B NMR** (128 MHz, CDCl$_3$): δ (ppm) = 22.0 (s, 1B, **B**(8)Cl), 0.1 (m, 2B, **B**H), –7.9 (m, 1B, **B**H), –12.6 (m, 4B, **B**H), –21.8 (m, 1B, **B**H); **^{11}B NMR** (128 MHz, CD$_3$CN): δ (ppm) = 21.4 (s, 1B, **B**(8)Cl), –1.3 (d, $^1J_{BH}$ = 122 Hz, 2B, **B**H), –9.1 (d, $^1J_{BH}$ = 132 Hz, 1B, **B**H), –13.2 (m, 4B, **B**H), –22.0 (m, 1B, **B**H); **MS** (HR-ESI, neg.) *m/z* = 786.2290 (calc.: 786.2261 [M+HCOOH–H]$^-$).

[3,3-{4-[1,1-Bis(4-hydroxyphenyl)but-1-en-2-yl]-2,2'-bipyridine-κ^2*N*,*N'*}-3-(CO)$_2$-*closo*-3,1,2-MoC$_2$B$_9$H$_{11}$] (10)

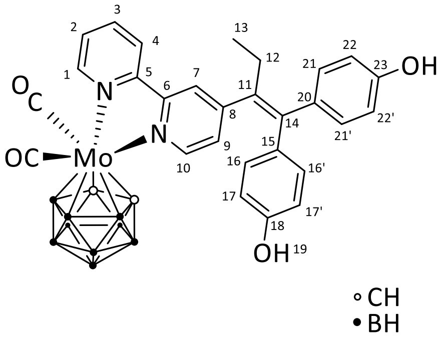

The same procedure was applied as for **8**, but using [NEt$_4$][3-(η^3-C$_3$H$_5$)-3-(CO)$_2$-*closo*-3,1,2-MoC$_2$B$_9$H$_{11}$] (**7**) (1.9 mL, 0.38 mmol, 1.0 equiv.) in THF (0.2 M), HCl in Et$_2$O (2 M in Et$_2$O, 0.22 mL, 0.46 mmol, 1.2 equiv.) and **6** (0.150 g, 0.38 mmol, 1.0 equiv.). The crude mixture was purified by flash column chromatography using ISOLERA FOUR on silica gel (SNAP Ultra 10g) with a CHCl$_3$/MeOH 1% → 12% MeOH (v/v) gradient. The first violet band was collected yielding **10** as a dark violet solid (0.89 g, 35%).

R$_F$ (CHCl$_3$/MeOH 10:0.7): 0.62; M(C$_{30}$H$_{33}$B$_9$MoN$_2$O$_4$) = 678.85 g mol^{-1}; **Elemental analysis**: C$_{30}$H$_{33}$B$_9$MoN$_2$O$_4$, calculated (%): C 53.08, H 4.90, N 4.13; found (%) C 52.78, H 5.38, N 3.75; **^{1}H NMR** (400 MHz, CD$_3$CN): δ (ppm) = 9.02 (d, $^3J_{HH}$ = 5.7 Hz, 1H, 1-C**H**), 8.70 (d, $^3J_{HH}$ = 6.0 Hz, 1H, 10-C**H**), 8.31 (d, $^3J_{HH}$ = 8.3 Hz, 1H, 4-C**H**), 8.26 (d, $^4J_{HH}$ = 1.6 Hz, 1H, 7-C**H**), 8.07 (ddd, $^3J_{HH}$ = 8.6, $^3J_{HH}$ = 7.7, $^4J_{HH}$ = 1.4 Hz, 1H, 3-C**H**), 7.51 (ddd, $^3J_{HH}$ = 7.7, $^3J_{HH}$ = 5.7, $^4J_{HH}$ = 1.8 Hz, 1H, 2-C**H**), 7.20 (dd, $^3J_{HH}$ = 6.0, $^4J_{HH}$ = 1.7 Hz, 1H, 9-C**H**), 7.10 (d, $^3J_{HH}$ = 8.6 Hz, 2H, 21,21'-C**H**), 7.06 (br s, 1H, 24-O**H**), 6.90 (br s, 1H, 19-O**H**), 6.84 (d, $^3J_{HH}$ = 8.6 Hz, 2H, 22,22'-C**H**), 6.75 (d, $^3J_{HH}$ = 8.6 Hz, 2H, 16,16'-C**H**), 6.51 (d, $^3J_{HH}$ = 8.6 Hz, 2H, 17,17'-C**H**), 2.70 (br s, 1H, C**H**$_{cluster}$), 2.62 (q, $^3J_{HH}$ = 7.6 Hz, 2H, 12-C**H$_2$**), 2.56 (br s, 1H, C**H**$_{cluster}$), 0.98 (t, $^3J_{HH}$ = 7.5 Hz, 3H, 13-C**H$_3$**), 0.70–3.61 (m, 9H, B**H**$_{cluster}$); **^{13}C{^{1}H} NMR** (101 MHz, CD$_3$CN) δ (ppm) = 260.3 (**C**O), 260.0 (**C**O), 157.4 (23-**C**$_{quat.}$), 157.0 (18-**C**$_{quat.}$), 156.5 (1-**C**H), 155.8 (6-**C**$_{quat.}$), 155.3 (10-**C**H), 154.6 (5-**C**$_{quat.}$), 153.8 (8-**C**$_{quat.}$), 144.4 (14-**C**$_{quat.}$), 139.7 (3-**C**H), 137.6 (11-**C**$_{quat.}$), 135.1 (20-**C**$_{quat.}$), 134.5 (15-**C**$_{quat.}$), 133.3 (16,16'-**C**H), 131.5 (21,21'-**C**H), 128.1 (9-**C**H), 126.5 (2-**C**H), 125.8 (7-**C**H), 124.6 (4-**C**H), 116.0 (22,22'-**C**H), 115.6 (17,17'-**C**H), 55.3 (**C**H$_{cluster}$), 28.7 (12-**C**H$_2$), 13.7 (13-**C**H$_3$); **^{11}B NMR** (128 MHz, CD$_3$CN): δ (ppm) = 7.2 (d, $^1J_{BH}$ = 120 Hz, 1B, **B**H), –3.2 (d, $^1J_{BH}$ = 130 Hz, 2B, **B**H), –6.3 (m, 1B, **B**H), –12.7 (m, 4B, **B**H), –17.2 (m, 1B, **B**H); **^{1}H NMR** (400 MHz, DMSO-*d$_6$*): δ (ppm) = 9.57 (s, 1H, 19-O**H**), 9.40 (s, 1H, 24-O**H**), 8.92 (d, $^3J_{HH}$ = 5.7 Hz, 1H, 1-C**H**), 8.82 (d, $^3J_{HH}$ = 8.1 Hz, 1H, 10-C**H**), 8.71 (d, $^4J_{HH}$ = 1.6 Hz, 1H, 7-C**H**), 8.57 (d, $^3J_{HH}$ = 6.1 Hz, 1H, 4-C**H**), 8.22 (ddd, $^3J_{HH}$ = 8.7, $^3J_{HH}$ = 7.5, $^4J_{HH}$ = 1.4 Hz, 1H, 3-C**H**), 7.69 (ddd, $^3J_{HH}$ = 7.3, $^3J_{HH}$ = 5.8, $^4J_{HH}$ = 1.3 Hz, 1H, 2-C**H**), 7.16 (dd, $^3J_{HH}$ = 6.1, $^4J_{HH}$ = 1.8 Hz, 1H, 9-C**H**), 7.02 (d, $^3J_{HH}$ = 8.5 Hz, 2H, 21,21'-C**H**), 6.79 (d, $^3J_{HH}$ = 8.5 Hz, 2H, 22,22'-C**H**), 6.67 (d, $^3J_{HH}$ = 8.6 Hz, 2H, 16,16'-C**H**), 6.46 (d, $^3J_{HH}$ = 8.6 Hz, 2H, 17,17'-C**H**), 2.91 (br s, 1H, C**H**$_{cluster}$), 2.76 (br s, 1H, C**H**$_{cluster}$), 2.63 (q, $^3J_{HH}$ = 7.3 Hz, 2H, 12-C**H$_2$**), 0.94 (t, $^3J_{HH}$ = 7.4 Hz, 3H, 13-C**H$_3$**), 0.70–3.61 (m, 9H, B**H**$_{cluster}$); **IR** (KBr): $\tilde{\nu}$ (cm^{-1}) = 3428 (s, ν(O–H)), 2958 (w, ν(C$_{alkyl}$–H)), 2927 (w, ν(C$_{alkyl}$–H)), 2869 (w, ν(C$_{alkyl}$–H)), 2526 (m, ν(B–H)), 1955 (s, ν(CO)), 1874 (s, ν(CO)), 1606 (m, ν(C=C)), 1508 (m, C$_{arom}$–H in-plane bending), 1475 (w, C$_{arom}$–H in-plane bending), 1435 (w, C$_{arom}$–H in-plane bending), 1381 (w), 1327 (w), 1259 (m, C$_{arom}$–O), 1169 (m, C$_{arom}$–O), 1169 (w), 1018 (w), 981 (w), 886 (m, C$_{arom}$–H out-of-plane bending), 787 (w, C$_{arom}$–H out-of-plane bending or ν(B–B), 745 (w), 590 (w), 568 (w), 530 (w), 471 (w); **MS** (HR-ESI, neg.) *m/z* = 678.231 (calc.: 678.228, [M–H]$^-$), 715.208 (calc.: 715.203, [M+Cl]$^-$).

2. X-ray Crystallography

CCDC-1944134 (**6**) and CCDC-1944251 (**9b**) contain the supplementary crystallographic data for this paper. These data can be obtained free of charge from The Cambridge Crystallographic Data Centre via www.ccdc.cam.ac.uk.

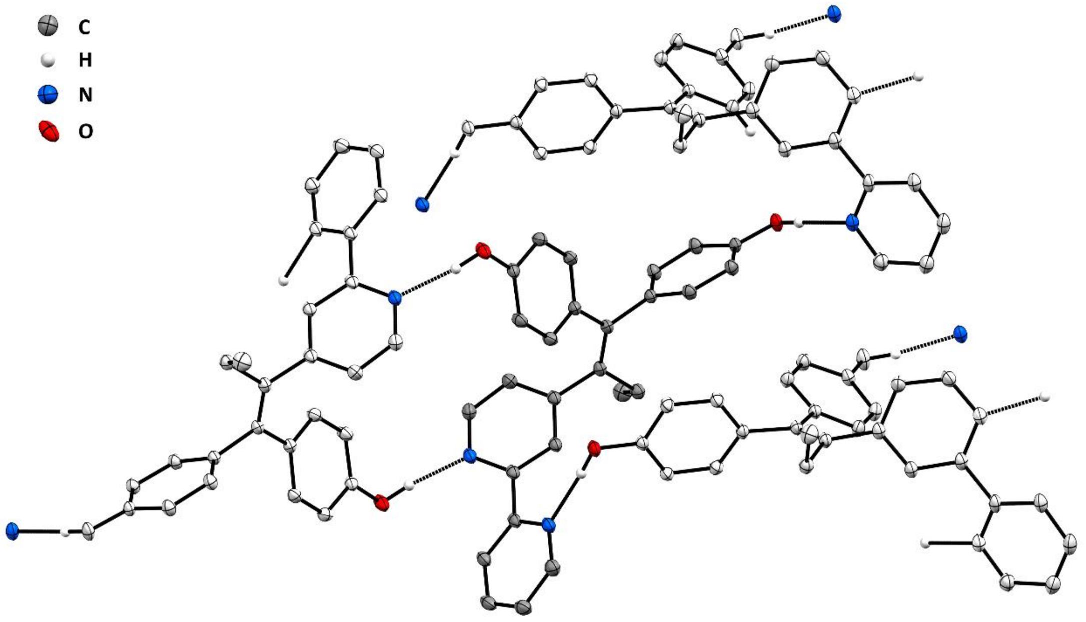

Figure S1. Packing of **6** in the solid state stabilized by intermolecular hydrogen bonds. The thermal ellipsoids are presented at 30% probability level and the hydrogen atoms not involved in hydrogen bonds are omitted for clarity. One molecule of **6** is highlighted.

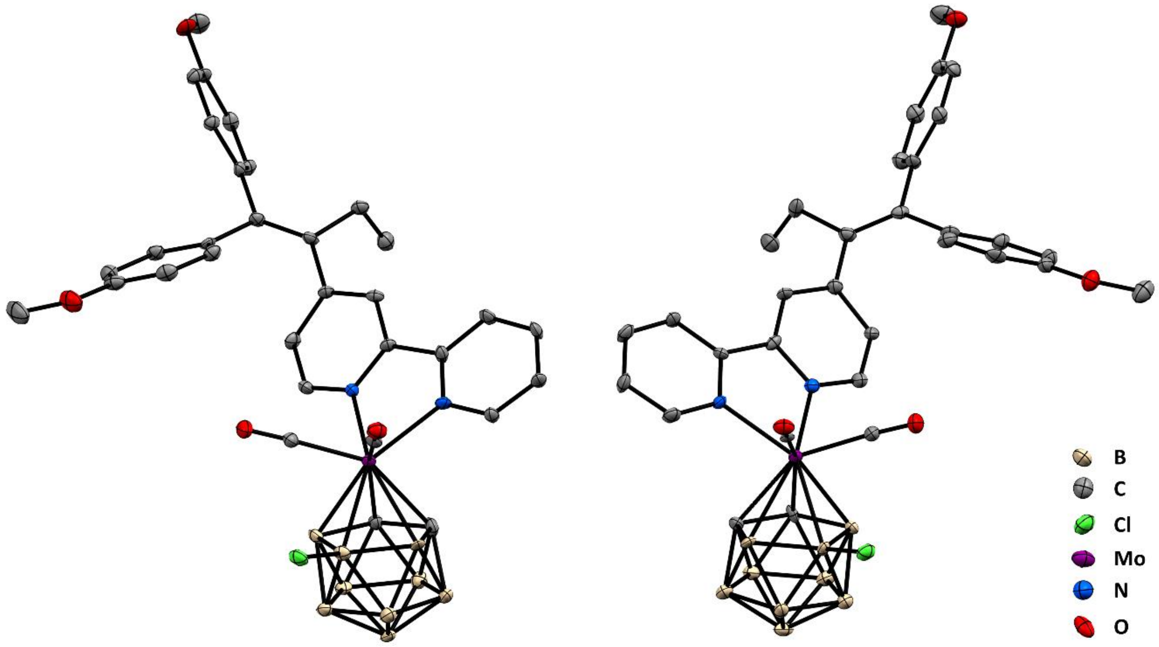

Figure S2. Molecular structures of the two isomers of **9b** in the solid state (not the packing in the crystal is shown here). Thermal ellipsoids are presented at 50% probability level and hydrogen atoms are omitted for clarity. CCDC number 1944251.

3. Stability and Solution Behavior Studies

3.1. NMR Spectroscopy

In the ^{1}H NMR spectra of the ligands alone (**3**, **5** and **6**) compared to the chemical shifts in complexes **8**, **9** and **10**, there is a clear coordination shift detectable (Figures S3−S5). The $CH_{cluster}$ signals split into two broad singlets due to the non-symmetric N,N-chelating ligand. Importantly, the coordination of **3**, **5** and **6** at molybdenum(II) generates always two isomers, which cannot be distinguished via NMR spectroscopy. The carbonyl groups give two slightly different signals at 255.3 and 254.5 ppm (**8**, CD_2Cl_2), 256.3 and 255.5 ppm (**9**, $CDCl_3$) or 260.3 and 260.0 ppm (**10**, CD_3CN) in the $^{13}C\{^1H\}$ NMR spectra of the respective complexes according to the C_1 symmetry of the whole molecule.

For *in vitro* cell culture tests, the stock solutions of sparingly water-soluble compounds are typically prepared in DMSO (ethanol or methanol are good alternatives) and stored below 4 °C. For that purpose, the chemical stability of **3**, **5**, **6** and **8−10** was tested in a solution of water-containing DMSO-d_6 in air for at least 36 days (**3**, **5**, **6**, **8**, **9**) and for 14 days for complex **10**. In all cases, ^{1}H and $^{11}B\{^1H\}$ NMR spectra, where appropriate, revealed that the ligands **3**, **5** and **6** can be stored in a DMSO stock solution for at least a month without decomposition, and the molybdacarboranes for 14 days up to a month with minor decomposition (where the decomposition products are the free ligands **3**, **5** or **6**, the *nido*-*ortho*-carborane ([*nido*-$C_2B_9H_{12}]^-$) and most likely a molybdenum species in higher oxidation state) (see Figures S6−S14).

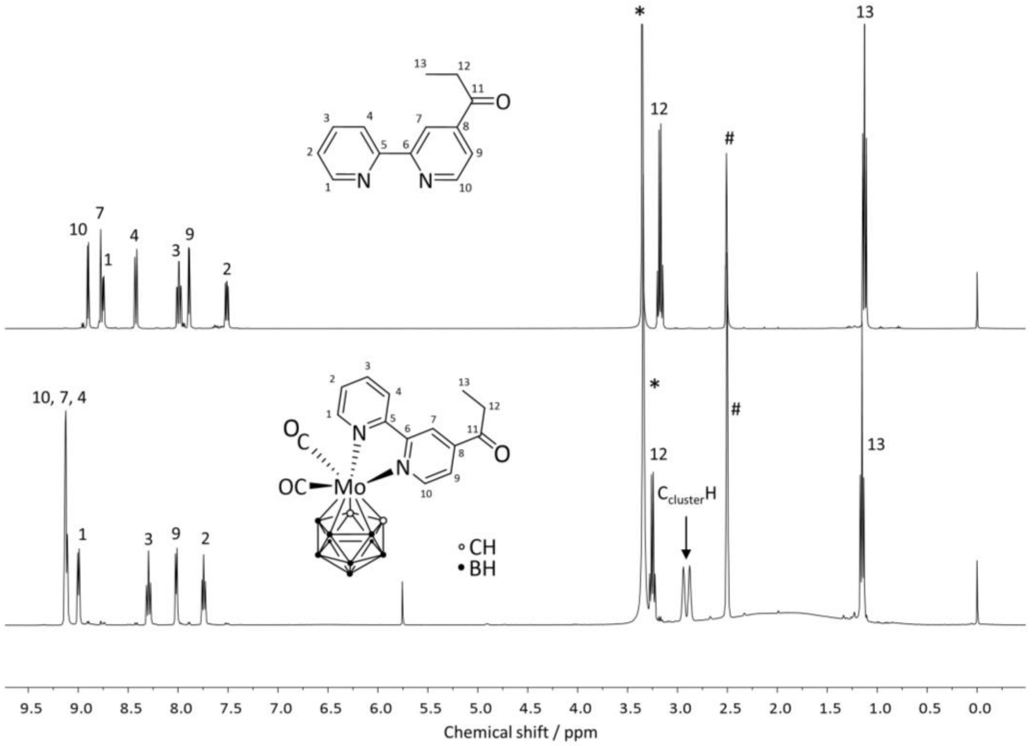

Figure S3. ^{1}H NMR spectra (DMSO-d_6) of free ligand (**3**, top) and the respective molybdacarborane complex (**8**, bottom). Clear coordination shift of the aromatic protons of the 2,2'-bipyridine unit (aromatic region) and a small shift of the ethyl group (aliphatic region). Additionally, for **8** the BH signals (0.66–3.71 ppm) and the CH protons of the carborane cluster split into two broad singlets (2.87 or 2.94 ppm) due to the non-symmetric ligand **3**. * Indicates the water residual peaks, # indicates the DMSO residual peak.

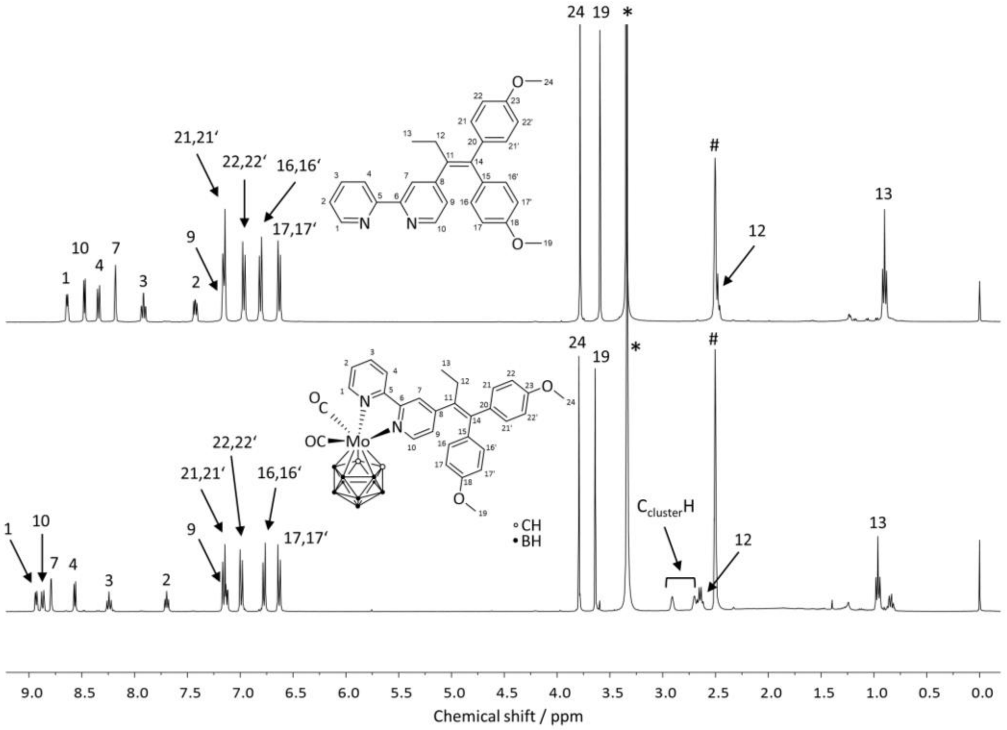

Figure S4. ^{1}H NMR spectra (DMSO-d$_6$) of free ligand (**5**, top) and the respective molybdacarborane complex (**9**, bottom). Clear coordination shift of the aromatic protons of the 2,2'-bipyridine unit (aromatic region) and a small shift of the 1,4-substituted phenyl rings and the ethyl group (aliphatic region). Additionally, for **9** the BH signals (0.71–3.21 ppm) and the CH protons of the carborane cluster split into two broad singlets (2.70 or 2.91 ppm) due to the non-symmetric ligand **5**. * Indicates the water residual peaks, # indicates the DMSO residual peak.

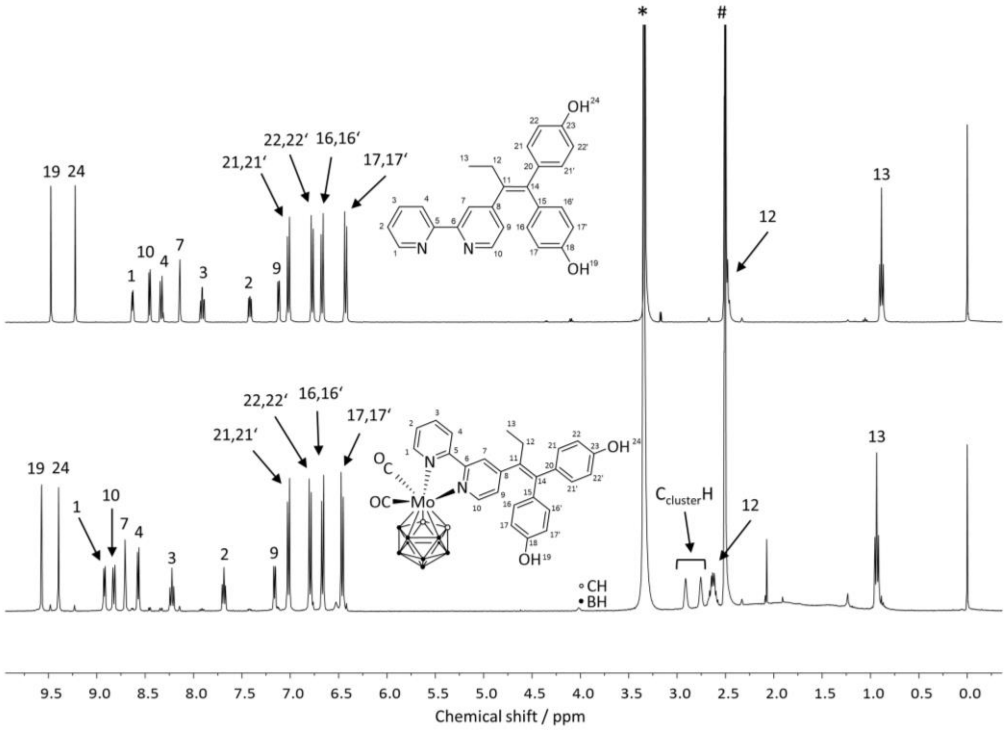

Figure S5. ^{1}H NMR spectra (DMSO-d$_6$) of free ligand (**6**, top) and the respective molybdacarborane complex (**10**, bottom). Clear coordination shift of the aromatic protons of the 2,2'-bipyridine unit (aromatic region) and a small shift of the 1,4-substituted phenyl rings and small shift of the ethyl group (aliphatic region). Also, in DMSO-d$_6$ the OH groups are detectable and shift upon coordination. Additionally, for **10** the BH signals (0.66–3.61 ppm) and the CH protons of the carborane cluster split into two broad singlets (2.76 or 2.91 ppm) due to the non-symmetric ligand **6**. * Indicates the water residual peaks, # indicates the DMSO residual peak.

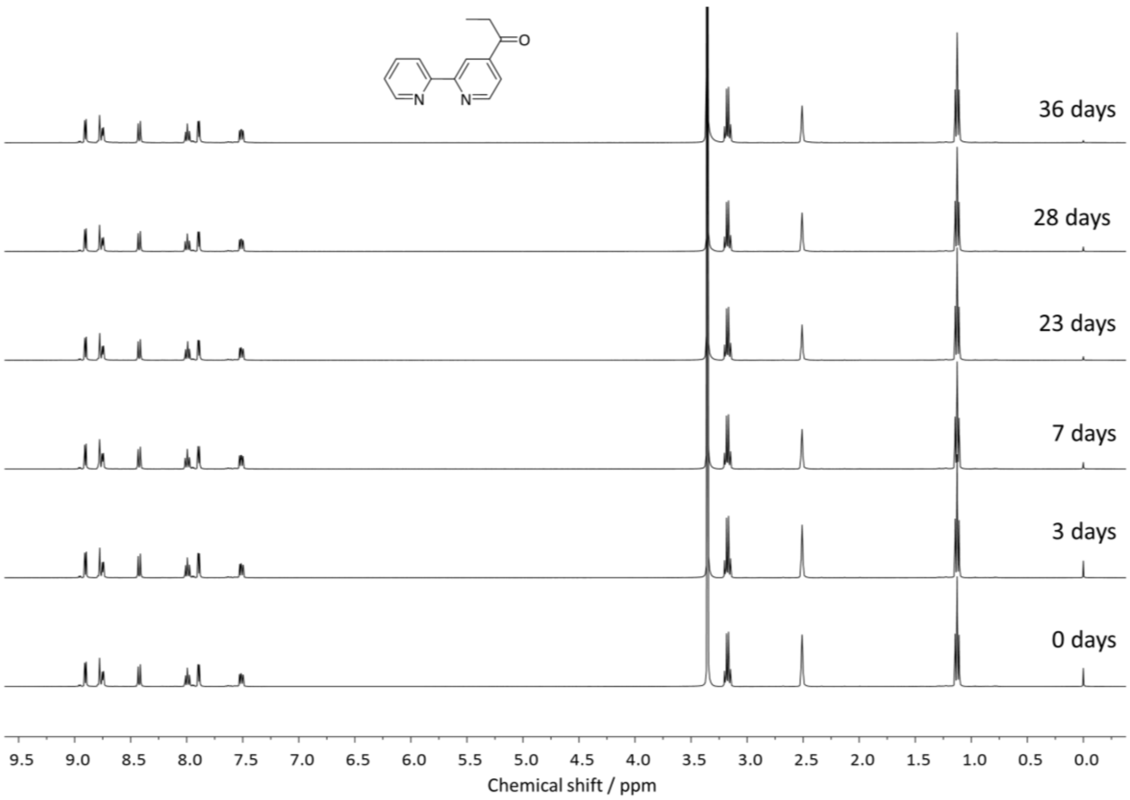

Figure S6. Stability test for **3** in DMSO-d_6 stock solution via ^1H NMR spectroscopy, over 36 days. No changes could be detected.

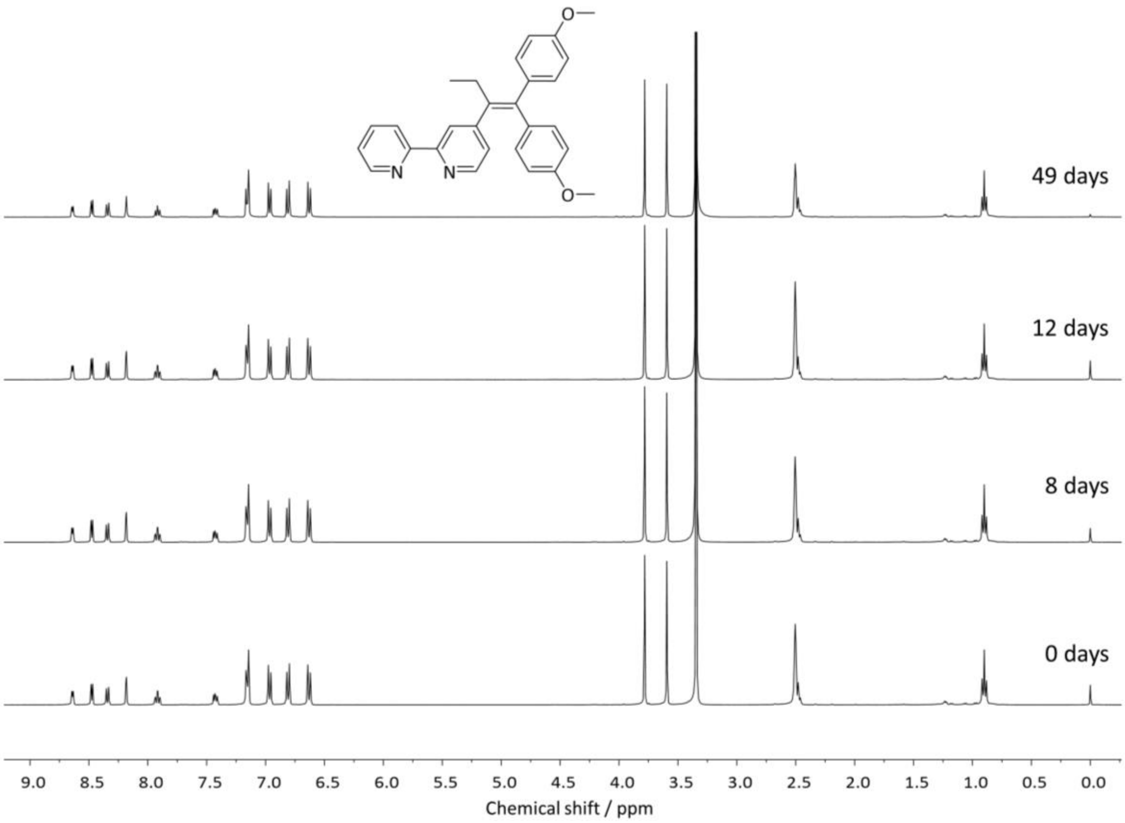

Figure S7. Stability test for **5** in DMSO-d_6 stock solution via ^1H NMR spectroscopy, over 49 days. No changes could be detected.

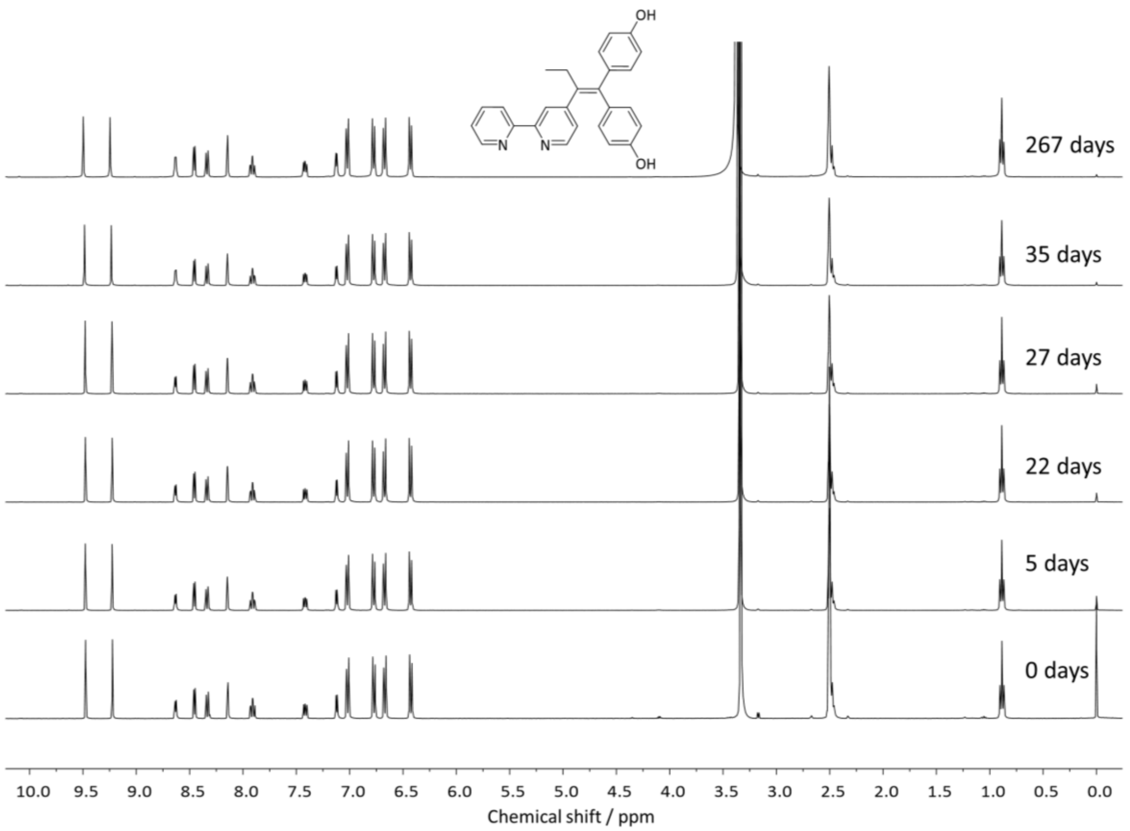

Figure S8. Stability test for **6** in DMSO-d_6 stock solution via ^{1}H NMR spectroscopy, over 267 days. No changes could be detected.

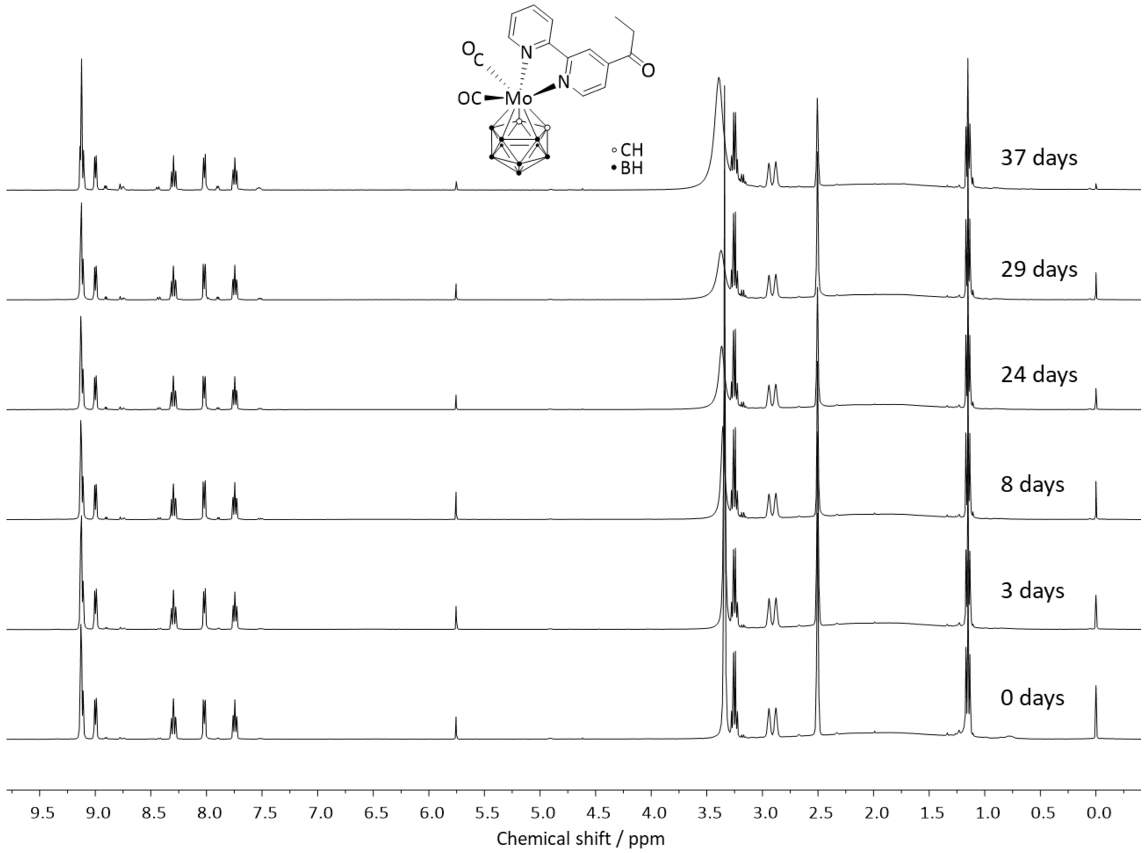

Figure S9. Stability test for **8** in DMSO-d_6 stock solution via ^{1}H NMR spectroscopy, over 37 days. Minor changes could be detected.

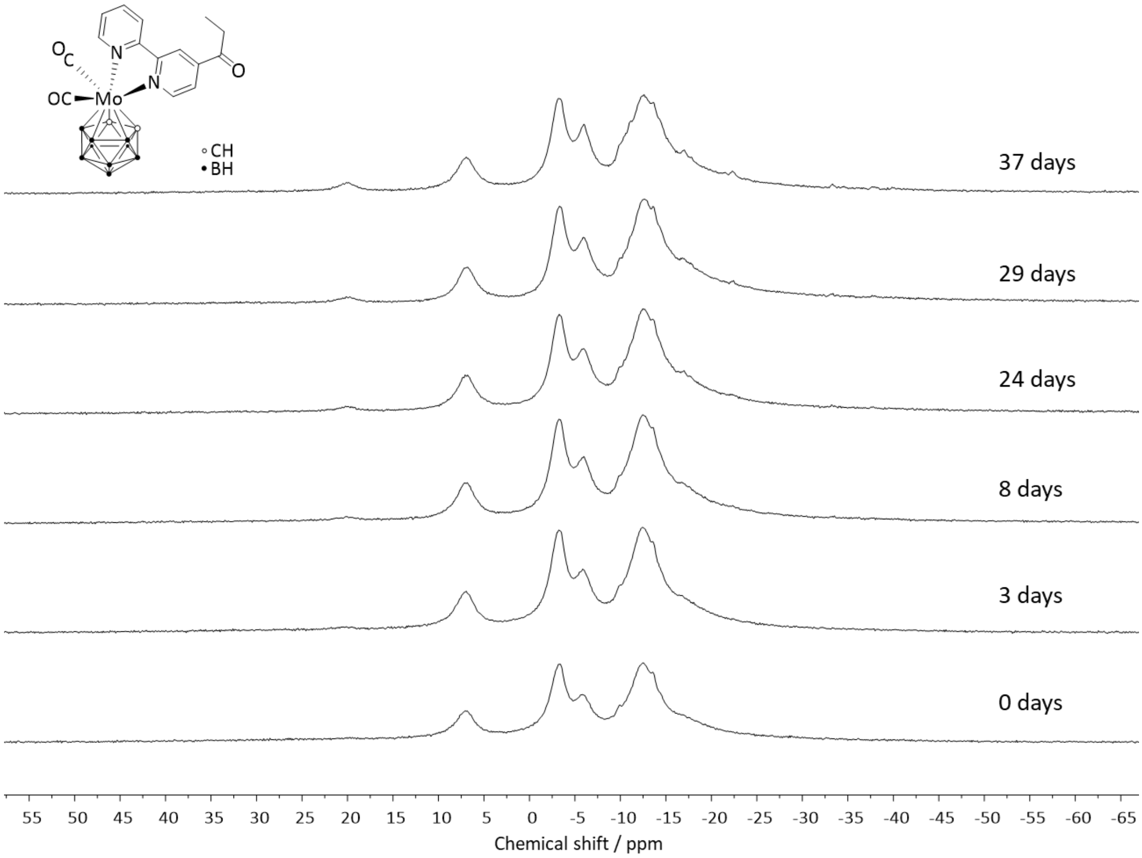

Figure S10. Stability test for **8** in DMSO-d_6 stock solution via $^{11}B\{^1H\}$ NMR spectroscopy, over 37 days. Minor changes could be detected. The slowly growing broad peak at ca. 20 ppm is due to agglomeration.

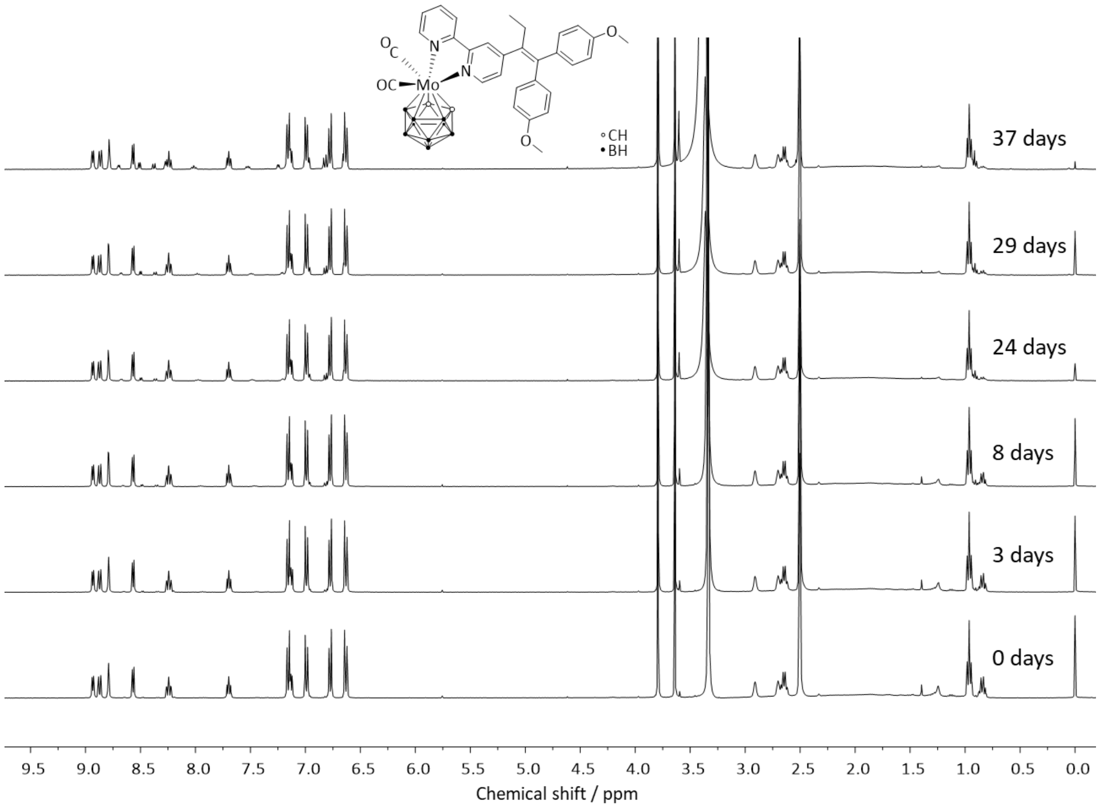

Figure S11. Stability test for **9** in DMSO-d_6 stock solution via ^1H NMR spectroscopy, over 37 days. Minor changes could be detected. Newly arising signals resemble the ones from the ligand **5** only.

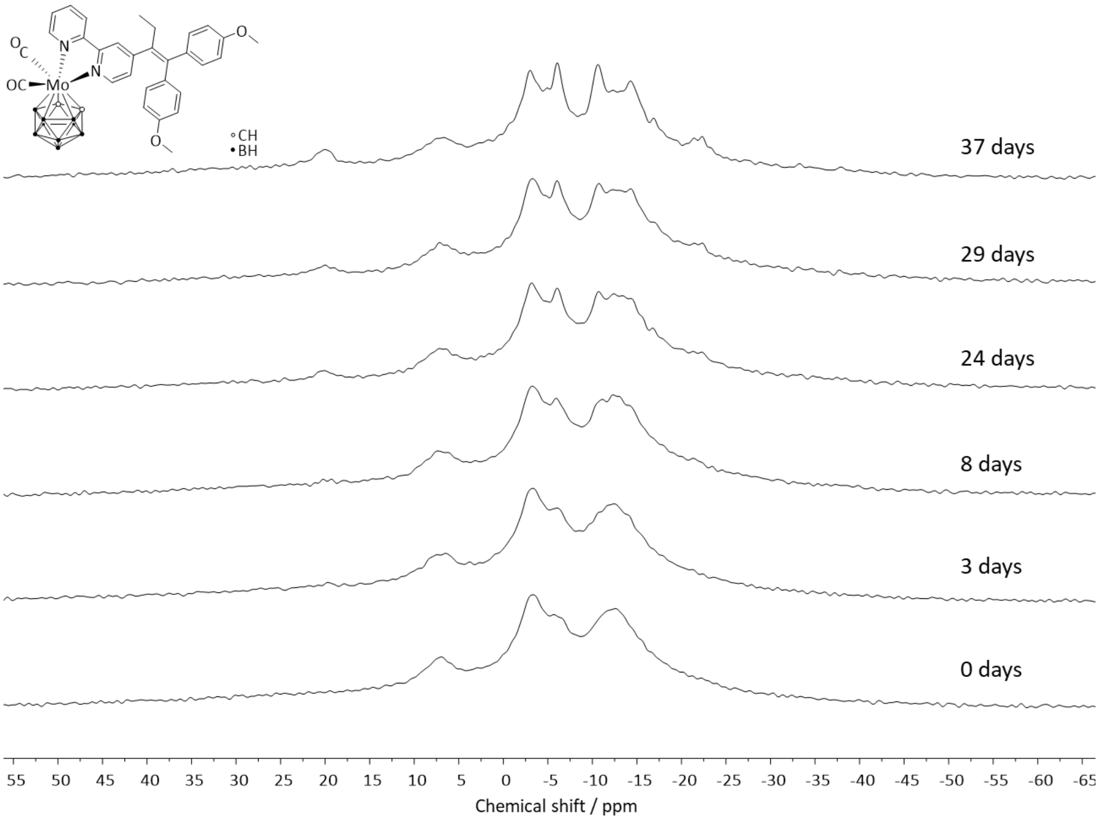

Figure S12. Stability test for **9** in DMSO-d$_6$ stock solution via $^{11}B\{^{1}H\}$ NMR spectroscopy, over 37 days. Minor changes could be detected. The slowly growing broad peak at ca. 20 ppm is due to agglomeration and the changes observed are not related to decomposition, but rather to reorganization of the molecules in solution.

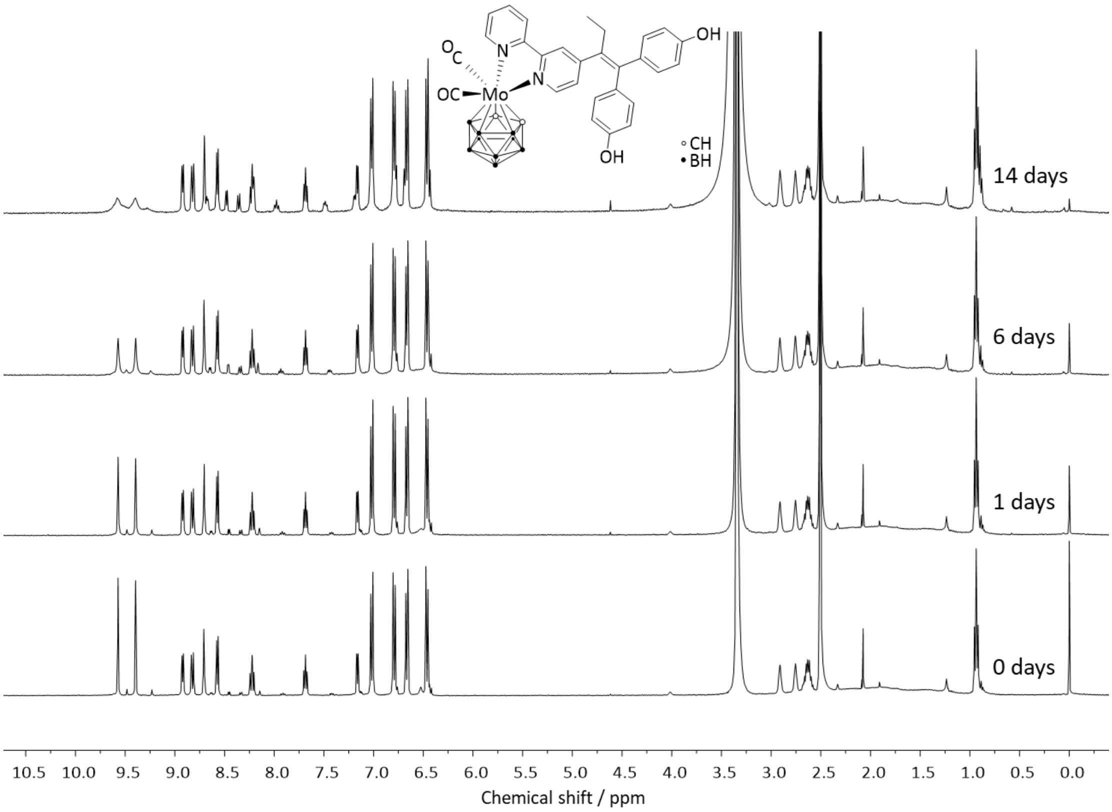

Figure S13. Stability test for **10** in DMSO-d$_6$ stock solution via ^{1}H NMR spectroscopy, over 14 days. Minor changes could be detected. Newly arising signals resemble the ones from the ligand **6** only. Hydroxy groups show H/D exchange.

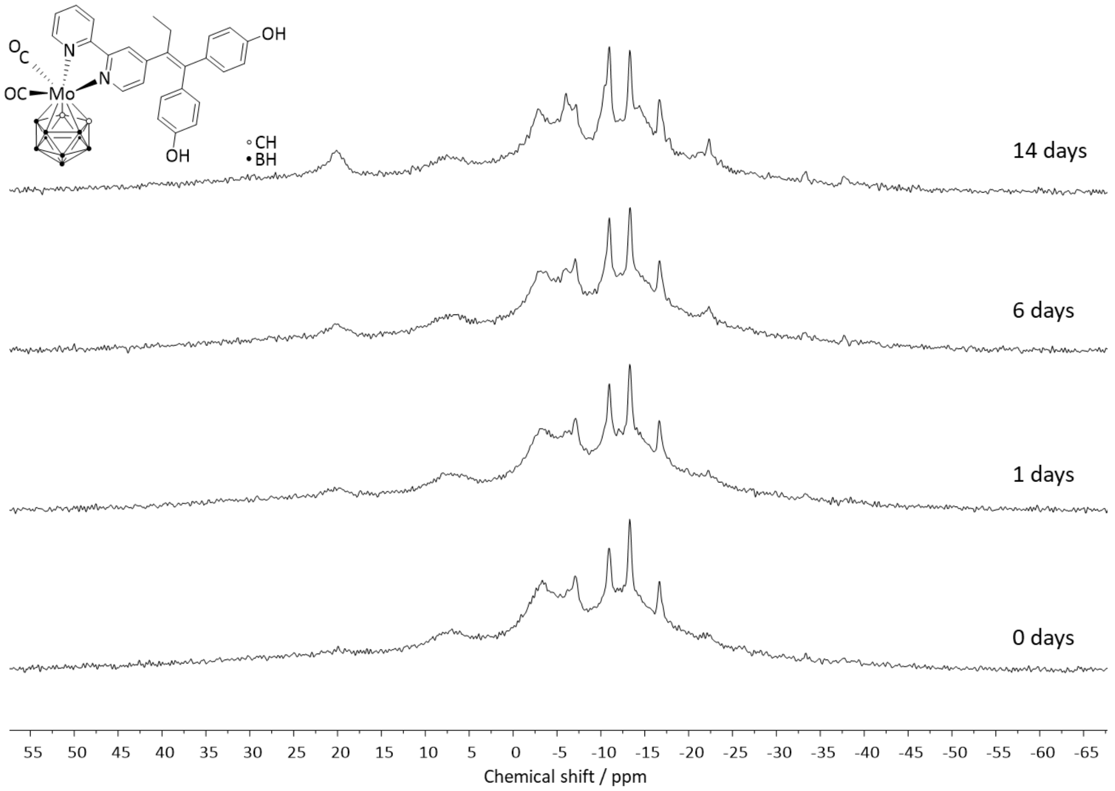

Figure S14. Stability test for **10** in DMSO-d_6 stock solution via $^{11}B\{^1H\}$ NMR spectroscopy, over 14 days. Minor changes could be detected. The slowly growing broad peak at ca. 20 ppm is due to agglomeration and the changes observed are not related to decomposition, but rather to reorganization of the molecules in solution.

3.2. UV-Vis, Fluorescence and Resonance Light Scattering (RLS) Spectroscopy

All working solutions were kept at a controlled temperature of 25 °C for the whole duration of the experiments. UV-Vis spectra were recorded at 25 °C, in the range of 190–800 nm, at 1.0 nm resolution. All measurements were corrected by subtracting the respective blank: PBS + 2.0 vol% DMSO for measurements in buffer. Fluorescence emission spectra were acquired in the range 290–550 nm, with cut-off (emission) filter at 290 nm. Three excitation wavelengths (λ_{exc}) were used, λ_{280} (Trp and Tyr), λ_{295} (Trp) and λ_{320} (reference λ_{exc} for [BSA_{noMg}–warfarin] complex), with excitation and emission slits of 5.0 and 2.5 (λ_{280}), 2.5 and 2.5 (λ_{295}), 5.0 and 2.5 (λ_{320}) nm, respectively. Each measurement was corrected subtracting the blank (PBS + 2 vol% DMSO) at the respective λ_{exc}. Raw data (number of scans = 3) were smoothed with 10 points moving average function, integrated in the software FLWINLab. The two- and three-components systems, i.e. [BSA_{noMg}–**6**/**10**], [BSA_{noMg}–site marker], [BSA_{noMg}–site marker–**6**/**10**] and [BSA_{noMg}–**6**/**10**–site marker], as well as the respective reference solutions were measured over 24 h with UV-Vis and fluorescence spectroscopy (spectra see Figure S15−S16). RLS spectra were measured after 1 h from sample preparation and were acquired with $\Delta\lambda = \lambda_{em} - \lambda_{exc} = 0$ nm, as reported,[17] closed slits, and 1% transmittance attenuator filter. Each measurement was corrected subtracting the blank (PBS + 2 vol% DMSO). Raw data (ns = 3) were smoothed with 10 points moving average function, integrated in the software FLWINLab.

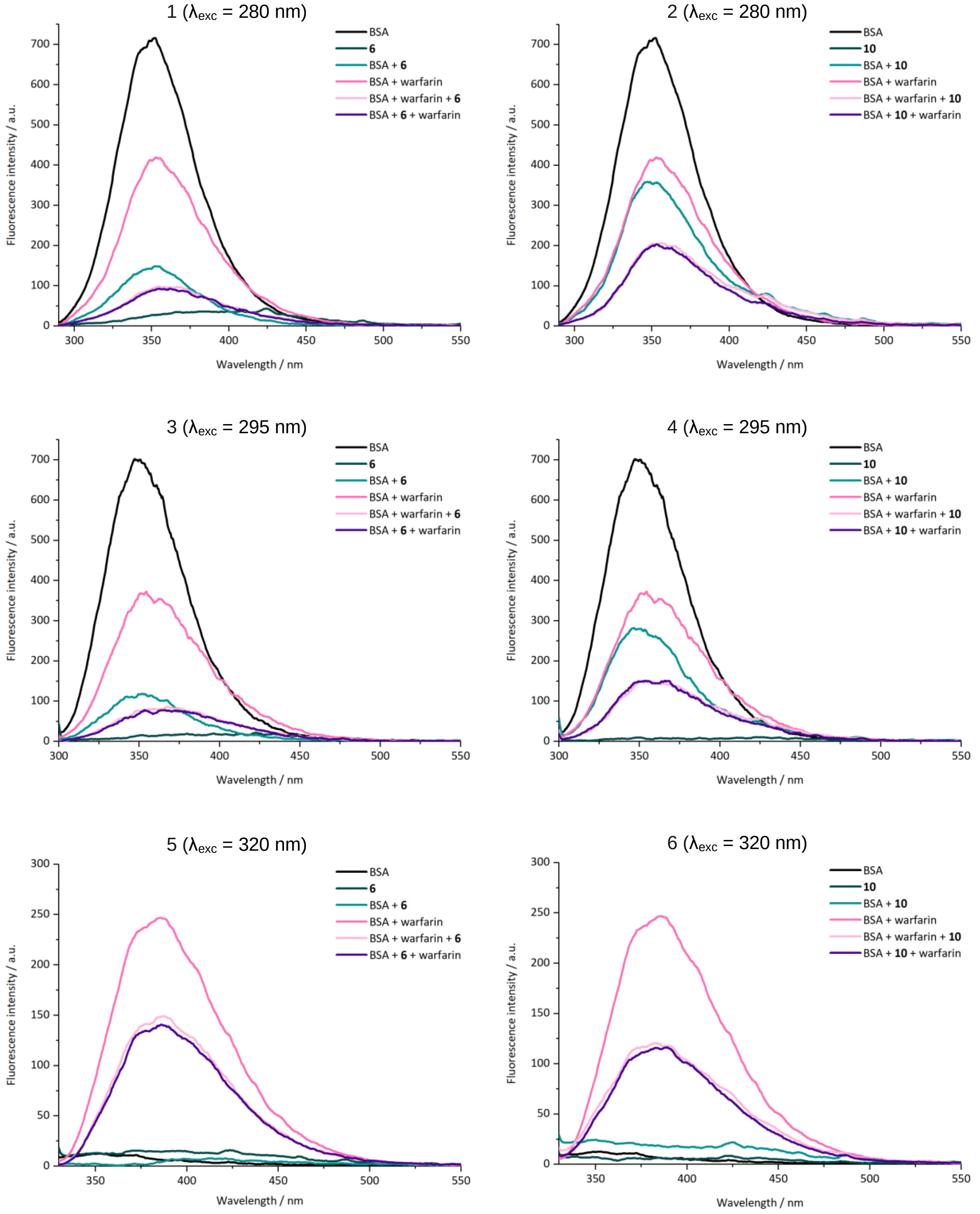

1 (λexc = 280 nm)
2 (λexc = 280 nm)
3 (λexc = 295 nm)
4 (λexc = 295 nm)
5 (λexc = 320 nm)
6 (λexc = 320 nm)
Fluorescence intensity / a.u.
Wavelength / nm
BSA
6
BSA + 6
BSA + warfarin
BSA + warfarin + 6
BSA + 6 + warfarin
10
BSA + 10
BSA + warfarin + 10
BSA + 10 + warfarin

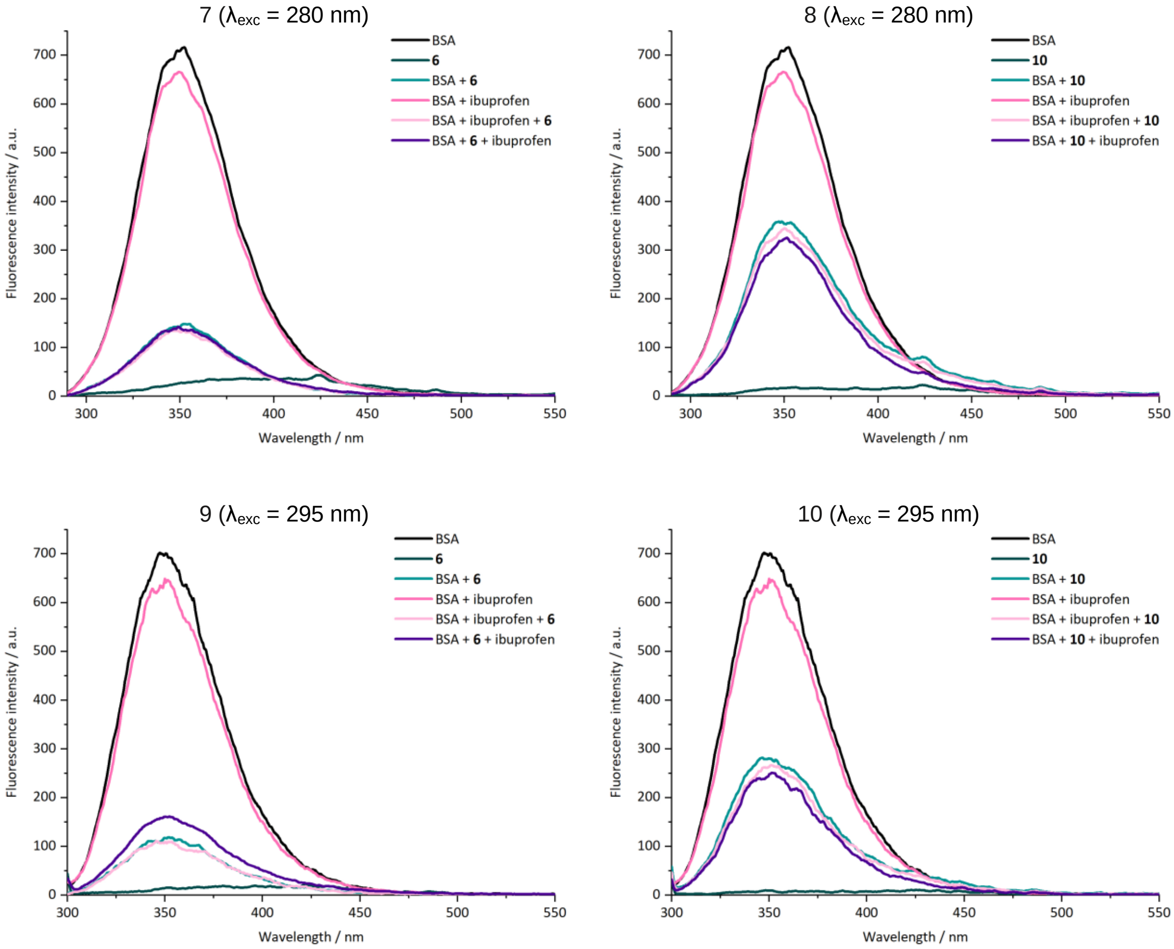

Figure S15. Fluorescence spectra of **6** and **10** with BSA_{noMg} and warfarin to investigate the binding to Sudlow's site I (panel 1−6) as well as with ibuprofen to investigate the binding to Sudlow's site II (panel 7−10). The same experiments are always shown for **6** and **10** in parallel for a direct comparison. Here, the order of the addition of the two components was investigated by comparison of the fluorescence quenching capacities.

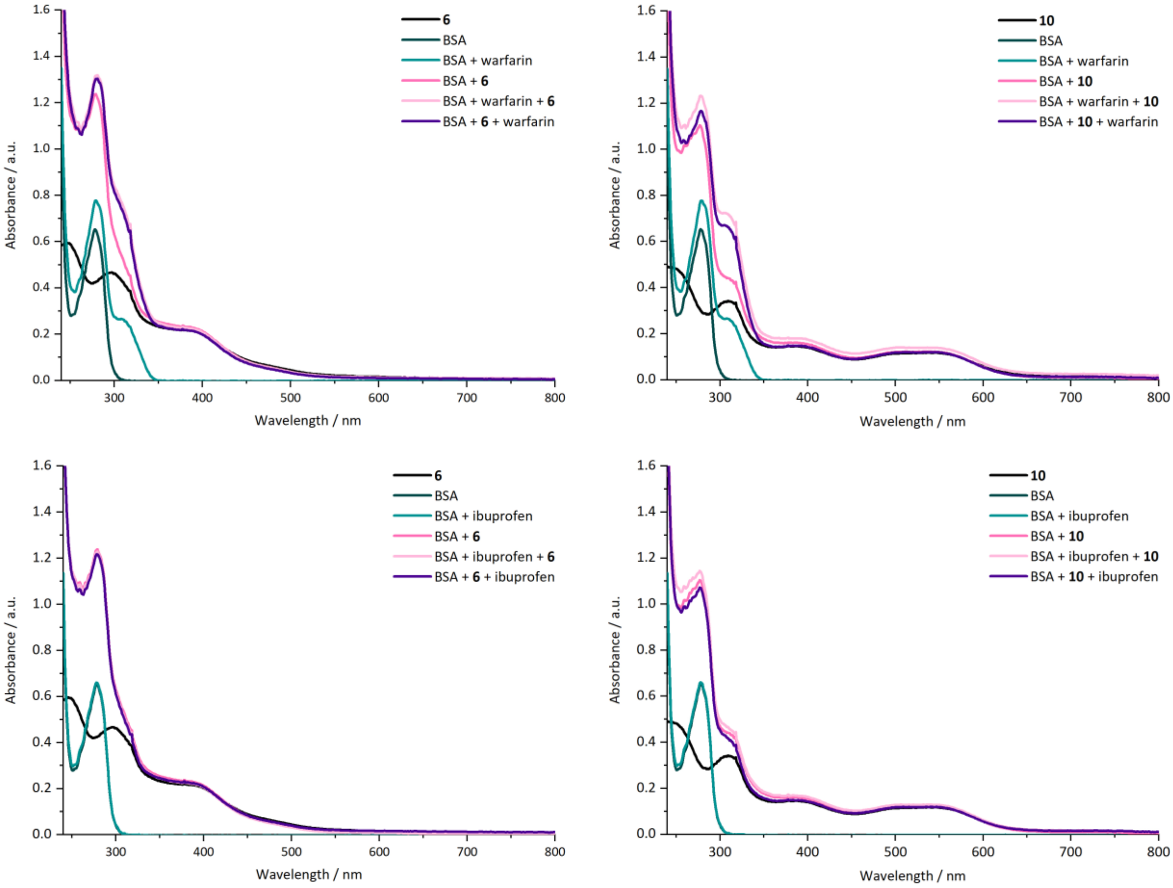

Figure S16. UV-Vis spectra of **6** and **10** with BSA_{noMg} and warfarin to investigate the binding to Sudlow's site I (top) as well as with ibuprofen to investigate the binding to Sudlow's site II (bottom). The same experiments are always shown for **6** and **10** in parallel for a direct comparison. Here, the order of the addition of the two components was investigated by comparison of the absorbance capacities.

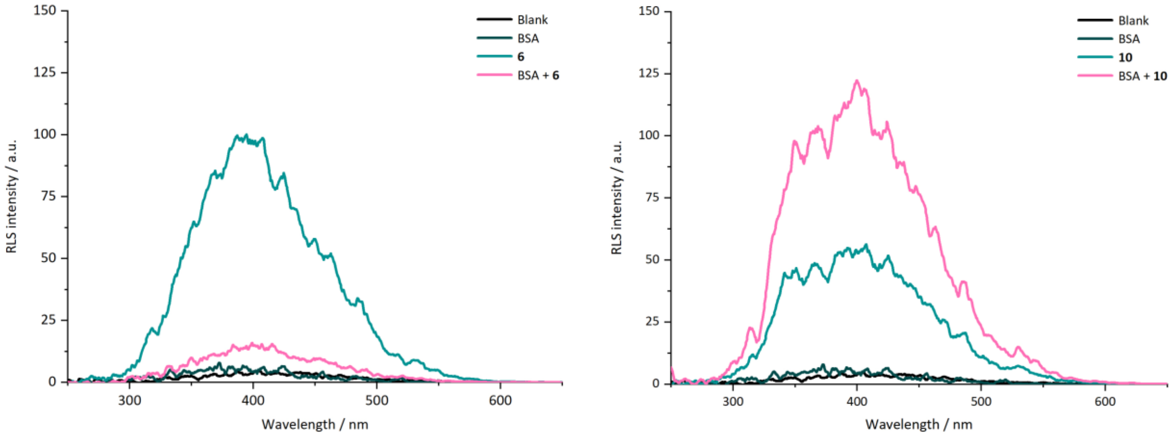

Figure S17. RLS profiles of **6** in PBS and BSA_{noMg} + **6** in PBS (left), **10** in PBS and BSA_{noMg} + **10** in PBS (right) as a measure of turbidity. BSA_{noMg} in PBS and PBS (blank) show very low RLS intensity. For the cases of **6**, the representation shows a clear improvement of the solubility of **6** as formulated with BSA_{noMg} (1:1). In contrast, for **10**, no improvement of the solubility could be detected as formulated with BSA_{noMg} (1:1), the RLS intensity even increases. The blanks consist of PBS + 2% DMSO.

3.3. Nanoparticle Tracking Analysis (NTA)

The possible self-assembling behavior of **6** and **10** in PBS/DMSO mixture (pH 7.4) was analyzed by Nanoparticle Tracking Analysis (NTA), with and without BSA_{noMg}, applying an analogous procedure as reported previously.[18] Samples of **6** and **10** in PBS/DMSO were prepared as described for UV-Vis, fluorescence and RLS measurements and measured 0.5−2 h after preparation. Samples of [BSA_{noMg}–**6**] and [BSA_{noMg}–**10**] were prepared as follows: The stock solution of BSA_{noMg} in PBS ($[BSA]_{PBS}$ = 1.27 mM) was diluted to final concentrations of 200 µM with PBS (V_{tot} = 5 mL), then the content of DMSO was preadjusted to 2 vol% (including the amount of DMSO from the stock solutions) before an aliquot of the stock solution of **6** or **10** (1.27 mM) was added, yielding a final concentration of the compounds of 20 µM. The solutions of [BSA_{noMg}–**6**] and [BSA_{noMg}–**10**] were measured at three time points, i.e. 1.5–2.2 h, 4–6.5 h and 20.5−22.25 h after preparation. For direct comparison, BSA_{noMg} in PBS/DMSO was measured as blank, using the same capture and processing parameters as for the respective [BSA_{noMg}–**6**] or [BSA_{noMg}–**10**] samples. All measurements were performed at 25 ± 0.1 °C. Each sample was measured in five independent captures (60 s/capture). Dilution was performed right before each measurement.

3.4. Mass Spectrometry

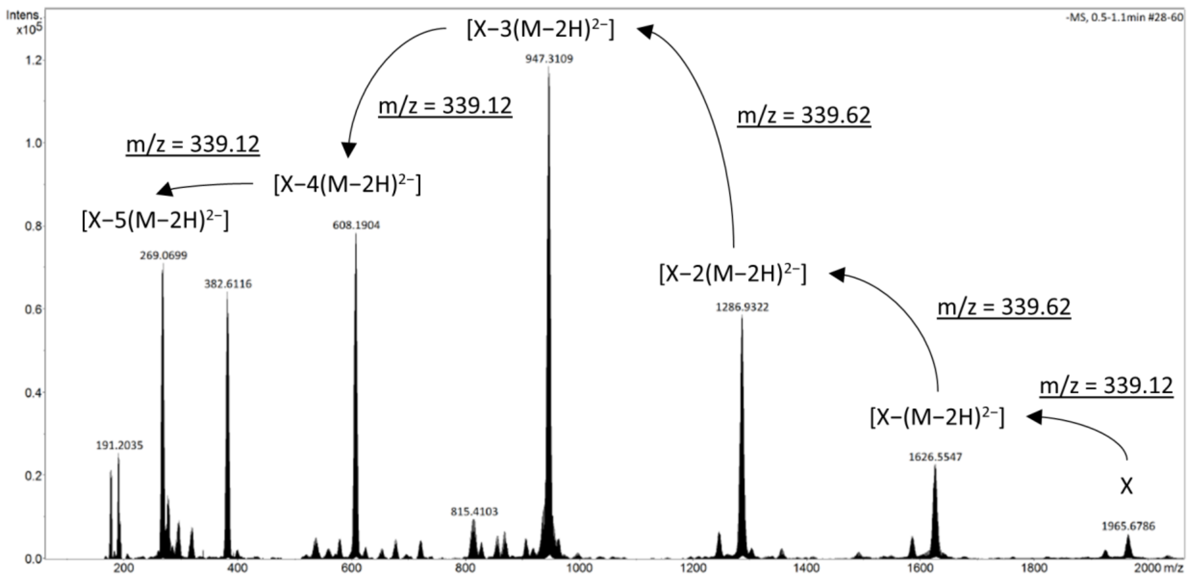

Figure S18. ESI high-resolution mass spectrum (neg.) of **10** showing the agglomeration taking place in solution (here: CH_3CN/CD_3CN).

4. Biological Studies

Reagents and cells. Fetal calf serum (FCS), phosphate-buffered saline (PBS), dimethyl sulfoxide (DMSO), 3-(4,5-dimethylthiazol-2-yl)-2,5-diphenyltetrazolium bromide (MTT), carboxyfluorescein-succinimidyl ester (CFSE), 3-methyl adenine (3-MA), crystal violet (CV) and acridine orange (AO) were obtained from Sigma Aldrich (St. Louis, MO, USA). 4′,6-diamidino-2-phenylindole (DAPI) was from Fluoromount-G (Southern Biotech., Birmingham, AL, USA). Cell culture medium RPMI-1640 was from Biowest (Riverside, MO, USA), while Dulbecco’s Modified Eagle’s/Ham’s Nutrient Mixture F-12 (DMEM/F-12) was from Biological Industries (Cromwell, CT, USA). Annexin V-FITC (AnnV) was from BD Pharmingen (San Diego, CA, USA), while ApoStat was bought from R&D Systems (Minneapolis, MN, USA). Dihydrorhodamin-123 (DHR 123) was from Thermo Fisher Scientific (Waltham, MA, USA). β-Galactosidase substrate FDG (FDG, Fluorescein digalactoside) and 4-amino-5-methylamino- 2',7'-difluorofluorescein diacetate (DAF-FM diacetate) from Molecular Probes (Eugene, OR, USA). Antiestrogen receptor alpha antibody (ab3575), antiestrogen receptor beta antibody (ab3576) and pre-

stained protein ladder – mid-range molecular weight (10−180 kDa) (ab116027) were purchased from Abcam plc. (Cambridge, UK). Cell lines (human breast adenocarcinoma MDA-MB-231, MDA-MB-361 and MCF-7; human malignant glioblastoma LN-229; glioma U-251) were purchased from the American Type Culture Collection (ATCC, Manassas, Virginia, USA). Cells were routinely cultivated in HEPES-buffered RPMI-1640 (MDA-MB-231, MDA-MB-361, MCF-7) or DMEM (U-251, LN-229) medium, supplemented with 10% FCS, 2 mM L-glutamine, 0.01% sodium pyruvate and antibiotics, at 37 °C in a humidified atmosphere with 5% CO_2. Peritoneal resident macrophages (Mf) were collected from C57BL/6 mice, obtained from the animal facility at the Institute for Biological Research "Siniša Stanković" (University of Belgrade), via peritoneal lavage with ice-cold PBS. Cells were counted and seeded in 96-well plates and incubated overnight, as described above. Before treatment, non-adherent cells were removed. Isolation of cells from animals was performed in accordance with local guidelines and approved by the local Institutional Animal Care and Use Committee (IACUC).

Preparation of drug solutions. DMSO stock solutions of **3, 6**, **8−10**, TAM-diOH, Fc-diOH were prepared at concentrations of 20 mM stored at −20 °C (**3**, **6**, TAM-diOH) or directly before usage (**8−10**, Fc-diOH). A stock solution of 3-MA was prepared according to the suppliers' data sheet. A stock solution of BSA_{noMg} in PBS was prepared at 22.56 mM concentration. For application to cell cultures, **3**, **6**, **8−10**, TAM-diOH or Fc-diOH (DMSO stock solution) were added to the BSA_{noMg} solution in PBS, with a 10:1 molar ratio of BSA_{noMg} over **3**, **6**, **8−10**, TAM-diOH or Fc-diOH, and incubated at room temperature for 0.5–1 h. Then the solutions were diluted with cell culture medium to the desired final concentrations (1.6, 3.1, 6.3, 12.5, 25, 50 or 100 μM). Final DMSO was 0.0078–0.5 vol%.

Colorimetric assays for cellular viability. Cells were exposed to various concentrations (0–100 μM) of **3, 6**, **8−10**, TAM-diOH or Fc-diOH for 72 h. For counting the number of attached (viable) cells, cells were fixed with 4% (w/w) paraformaldehyde for 10 min at room temperature, and subsequently stained for 15 min with 2% crystal violet (CV) solution. Cells were then washed with tap water, dried in air, and the CV dye was dissolved in 33% (w/w) acetic acid solution. For the detection of mitochondrial respiration, cells were cultivated in MTT staining solution (0.5 mg mL^{-1}) for approximately 1 h. The dye was then discarded, and the formed formazan (purple) was dissolved in DMSO. The absorbance was measured with an automated microplate reader at λ_{max} = 540 nm, with the reference λ_{max} = 670 nm. Cells treated only with BSA_{noMg} and DMSO (control) were also measured, as reference for BSA-containing drug formulations. Cell viability is expressed as percentage (%) relative to control and presented as mean ± SD. Experiments were run in three independent replicates.

Flow cytometry. For better insights into the mechanisms of action of **6** and **10** compared to Fc-diOH, MCF-7 cells were incubated with **6**, **10** or Fc-diOH at the respective IC_{50} dose and analyzed by flow cytometry. Several staining protocols were carried out in parallel, in independent experiments: i) AnnV/PI for the detection of apoptotic cell death; ii) AO for the detection of acidic vacuoles; iii) ApoStat for checking caspase activity; iv) CFSE for detecting interference with cellular proliferation; v) DHR 123 for the detection of reactive oxygen/nitrogen species (ROS/RNS) levels; vi) β-galactosidase assay for measuring senescence-associated (SA) β-galactosidase activity; vii) DAF-FM diacetate for measurement of intracellular nitric oxide (NO) quantity. Cells treated only with BSA_{noMg} (control) were also measured, as reference for BSA_{noMg}-containing drug formulations. For AnnV/PI, AO and ApoStat staining, cells were treated with **6**, **10** or Fc-diOH, and then detached and washed with PBS. Cells were then stained, according to the manufacturer's protocols, with AnnV/PI (15 min, room temperature), or ApoStat (30 min, 37 °C), or AO (10 μM; 15 min, 37 °C), in PBS. Finally, cells were washed, resuspended in PBS (or AnnV-binding buffer for AnnV/PI), and analyzed. For CFSE staining, cells were first treated with a PBS solution of CFSE (1 μM; 10 min, 37 °C), then washed, seeded and then exposed to **6**, **10** or Fc-diOH. Cells were then washed, trypsinized, dissolved in PBS and analyzed. For DAPI staining, cells were cultivated on chamber slides overnight, and then treated with **6**, **10** or Fc-diOH. Cells were then fixed with 4% (w/w) paraformaldehyde (15 min, room temperature), and the chamber slides were stained with DAPI (2 min). Finally, cells were washed with PBS and prepared for fluorescence microscopy by layering with fluorescent mounting medium (Dako, Glostrup, Denmark). For the measurement of reactive oxygen/nitrogen species (ROS/RNS), cells were stained with 1 μM dihydrorhodamin-123 (DHR 123) for 20 min at 37 °C, and then exposed to an IC_{50} dose of the experimental compounds for 72 h. At the end of cultivation, cells were washed, detached and

analyzed with CyFlow® Space Partec using the PartecFloMax® software. For the measurement of the intracellular nitric oxide (NO) quantity, cells were treated with an IC_{50} dose of the experimental compounds for 72 h, washed, trypsinized and stained with 5 µM 4-amino-5-methylamino- 2',7'-difluorofluorescein diacetate (DAF-FM diacetate) for 1 h at 37 °C in phenol red-free RPMI 1640. Thereafter, cells were washed and additionally incubated for 15 min in fresh RPMI 1640 without phenol red and serum, to finish the reaction of de-esterification. Analysis was done as indicated for DHR 123. For the determination of the β-galactosidase activity, cells were treated with an IC_{50} dose of **6**, **10** or Fc-diOH for 72 h, and then stained with β-galactosidase substrate FDG (fluorescein-di-β-D-galactopyranoside) to a final concentration of 1 mM. After 1 min incubation at 37 °C, cells were analyzed by flow cytometry as described. Channels FL1 (green emission), FL2 (orange emission) and/or FL3 (dark red emission) were used for fluorescence detection, according to the specific staining agent. Experiments were run in three independent replicates.

Fluorescence microscopy. For DAPI staining, cells were cultivated on chamber slides overnight, then treated with **6**, **10** or Fc-diOH. Cells were then fixed with 4% (w/w) paraformaldehyde (15 min, room temperature), and the chamber slides were covered with DAPI Fluoromount-G (Southern Biotech, Birmingham, AL, USA) before analysis. The slides were analyzed with a Zeiss AxioObserver Z1 inverted fluorescence microscope (Carl Zeiss AG, Oberkochen, Germany) at 200× magnification. Morphological signs of apoptosis (irregular nuclei shape, condensed chromatin, apoptotic bodies) were examined in three independent experiments.

Western blot. Cells were lyzed in protein lysis buffer composed of 62.5 mM Tris-HCl pH 6.8, 2% (w/v) SDS, 10% glycerol and 50 mM dithiothreitol. The protein content was measured by Lowry's method. Proteins were electrophoresed on 12% SDS-polyacrylamide gel. Afterwards, proteins were transferred onto a polyvinylidenedifluoride (PVDF) membrane using a semidry blotting system (Fastblot B43; BioRad, Göttingen, Germany). Membranes were blocked with 5% BSA in PBS with 0.1% Tween 20 for 1 h at room temperature. Then, membranes were incubated overnight at 4 °C with specific antibodies: antiestrogen receptor alpha antibody, antiestrogen receptor beta antibody (Abcam, Cambridge, UK) and β-actin (Sigma Aldrich, St. Louis, MO, USA). As a secondary antibody goat anti-rabbit IgG-HRP and bovine anti-mouse IgG-HRP (Santa Cruz Biotechnology, Dallas, TX, USA) were used. Dilutions for primary antibodies were 1:1000, for secondary 1:3000. Bands were detected with a chemiluminescence detection system (ECL; GE Healthcare, Chalfont St. Giles, Buckinghamshire, UK).

Statistical analysis. Analysis of variance (ANOVA) followed with a Students−Newman−Keuls test was used for significance of the differences between treatments, and a p value less than 0.05 was taken as statistically significant.

5. Cell Viability

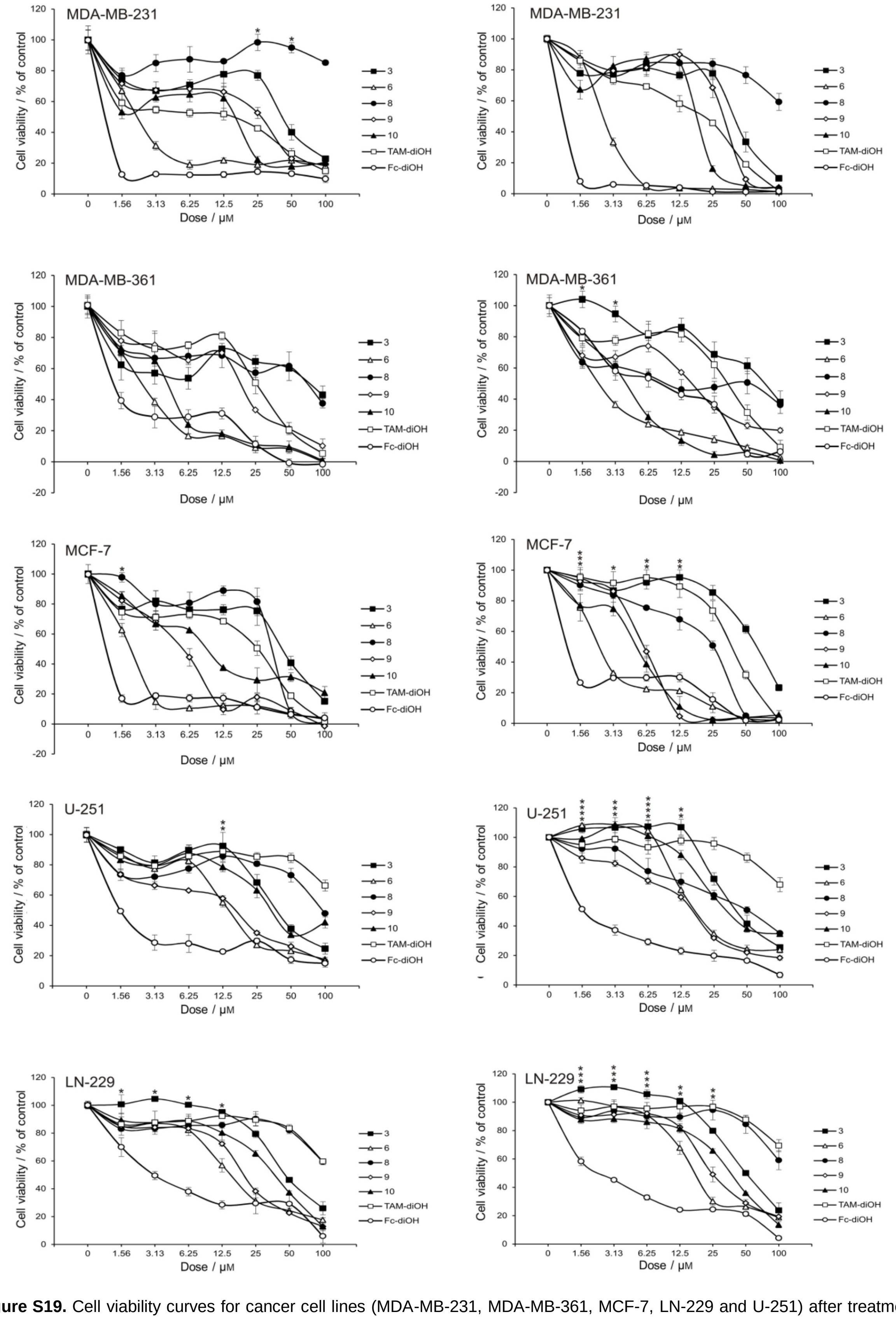

Figure S19. Cell viability curves for cancer cell lines (MDA-MB-231, MDA-MB-361, MCF-7, LN-229 and U-251) after treatment (72 h) with **3**, **6**, **8**–**10**, TAM-diOH and Fc-diOH obtained from MTT (left panel) and CV (right panel) assays. Concentration range varied from 1 to 100 µM for **3**, **6**, **8**–**10**, TAM-diOH and Fc-diOH. Data are expressed as % viable cells with respect to the control, as mean value from three independent experiments. Standard deviations are also shown. * indicates values which are <u>not</u> statistically significant ($p < 0.05$), with respect to the control.

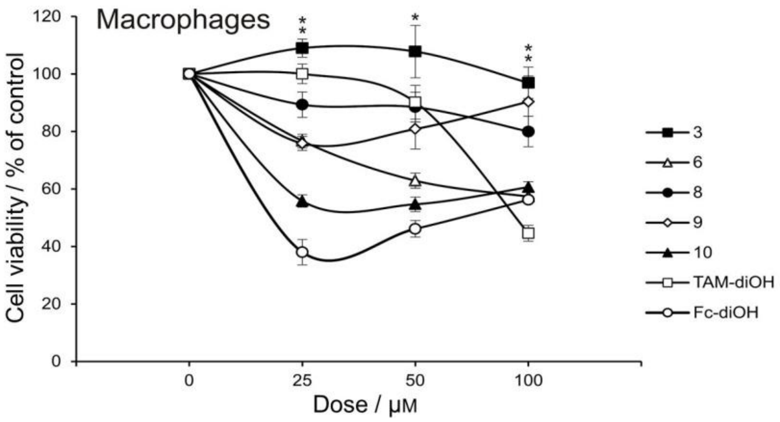

Figure S20. Cell viability curves for mouse macrophages (Mf) after treatment (72 h) with **3**, **6**, **8**–**10**, TAM-diOH and Fc-diOH obtained from CV assays. Concentration of **3**, **6**, **8**–**10**, TAM-diOH and Fc-diOH was 25, 50 or 100 μM. Standard deviations are also shown. * indicates values which are <u>not</u> statistically significant ($p < 0.05$), with respect to the control.

In order to draw interferences between the activity of the novel molecules, which are inspired by tamoxifen, a drug for hormone-dependent breast cancer treatment, and the presence of the ER receptors, the receptor status for ER-α and ER-β was tested. This is reasonable, due to the fact that the two transcriptions factors (ER-α and ER-β) have a very important role in health and disease, and the real ER status of the tested cell lines should always be doublechecked to draw a connection to the tested compounds.[19]

Therefore, bridging the gap between the tested cell lines and the estrogen receptors alpha (ER-α) and beta (ER-β), western blot analysis was performed (Figure S21). It revealed that the tested cell lines express the ER-α – MDA-MB-361, MCF-7 and LN-229 in higher amounts, MDA-MB-231 in medium amounts and U-251 to a lesser extent. Concerning the expression of the ER-β, also all cell lines express this receptor, but to varying extent. MDA-MB-231 and U-251 show low expression of ER-β, whereas MDA-MB-361, MCF-7 and LN-229 express higher amounts of ER-β. Vivid discussion is found in the literature about the validity of the commercially available ER-α/ER-β antibodies: their selective binding is doubted, and in turn, the estrogen receptor status of the cells is found to be contradicting throughout the literature.[20] However, it seems that the anticancer potential of our newly designed compounds is not in correlation with ER receptor expression, under the applied concentrations, indicating the existence of other targets for these molecules.

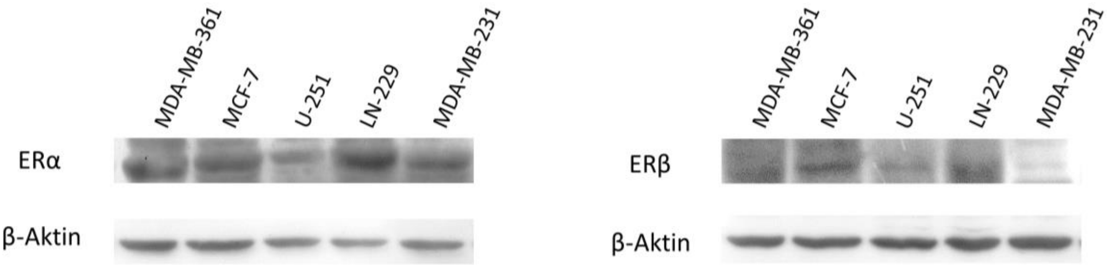

Figure S21. Estrogen receptor alpha (ER-α) (left) and beta (ER-β) (right) status of the tested cell lines, namely MDA-MB-361, MCF-7, U-251, LN-229 and MDA-MB-231. ER-α – detected band of ca. 64 kDa; ER-β – detected band of ca. 55 kDa; β-Aktin – detected band of 42 kDa.

6. References

[1] G. Jaouen, S. Top, A. Vessières, G. Leclercq, J. Quivy, L. Jin, A. Croisy, *C.R. Acad. Sci. Paris Ser. IIc* **2000**, 89.
[2] D. D. Yu, B. M. Forman, *J. Org. Chem.* **2003**, *68*, 9489.
[3] S. J. Dossett, S. Li, F. G. A. Stone, *J. Chem. Soc., Dalton Trans.* **1993**, 1585.
[4] T. N. Y. Hoang, M. Humbert-Droz, T. Dutronc, L. Guénée, C. Besnard, C. Piguet, *Inorg. Chem.* **2013**, *52*, 5570.
[5] S. O. Mihigo, W. Mammo, M. Bezabih, K. Andrae-Marobela, B. M. Abegaz, *Bioorg. Med. Chem.* **2010**, *18*, 2464.
[6] R. K. Harris, E. D. Becker, S. M. Cabral De Menezes, R. Goodfellow, P. Granger, *Concepts Magn. Reson.* **2002**, *14*, 326.
[7] CrysAlis Pro: Data collection and data reduction software package, Rigaku Oxford Diffraction.
[8] SCALE3 ABSPACK: Empirical absorption correction using spherical harmonics.
[9] G. M. Sheldrick, *Acta Crystallogr. A* **2015**, *71*, 3.
[10] C. B. Hübschle, G. M. Sheldrick, B. Dittrich, *J. Appl. Cryst.* **2011**, *44*, 1281.
[11] G. M. Sheldrick, *Acta Crystallogr. A* **2008**, *64*, 112.
[12] C. F. Macrae, P. R. Edgington, P. McCabe, E. Pidcock, G. P. Shields, R. Taylor, M. Towler, J. van de Streek, *J. Appl. Cryst.* **2006**, *39*, 453.
[13] a) E. F. Pettersen, T. D. Goddard, C. C. Huang, G. S. Couch, D. M. Greenblatt, E. C. Meng, T. E. Ferrin, *J Comput. Chem.* **2004**, *25*, 1605; b) Persistence of Vision Pty. Ltd. (2004), Persistence of Vision Raytracer (Version 3.6).
[14] K. E. Henegar, S. W. Ashford, T. A. Baughman, J. C. Sih, R.-L. Gu, *J. Org. Chem.* **1997**, *62*, 6588.
[15] Y.-Q. Fang, O. Lifchits, M. Lautens, *Synlett* **2008**, *2008*, 413.
[16] S. Kumar, M.-T. Ho, Y.-T. Tao, *Org. Lett.* **2016**, *18*, 200.
[17] a) R. F. Pasternack, P. J. Collings, *Science* **1995**, *269*, 935; b) R. F. Pasternack, C. Bustamante, P. J. Collings, A. Giannetto, E. J. Gibbs, *J. Am. Chem. Soc.* **1993**, *115*, 5393.
[18] B. Schwarze, M. Gozzi, C. Zilberfain, J. Rüdiger, C. Birkemeyer, I. Estrela-Lopis, E. Hey-Hawkins, *J. Nanoparticle Res.* **2019**, accepted manuscript.
[19] a) M. Jia, K. Dahlman-Wright, J.-Å. Gustafsson, *Best Pract. Res. Clin. Endocrinol. Metab.* **2015**, *29*, 557; b) J. Hartman, A. Ström, J.-A. Gustafsson, *Steroids* **2009**, *74*, 635; c) C. Palmieri, G. J. Cheng, S. Saji, M. Zelada-Hedman, M. Wärri, Z. Weihua, S. van Noorden, T. Wahlstrom, R. C. Coombes, M. Warner et al., *Endocr. Relat. Cancer* **2002**, *9*.
[20] a) G. C. Chamness, W. D. Mercer, W. L. McGuire, *J. Histochem. Cytochem.* **1980**, *28*, 792; b) M. K. Gandhari, C. R. Frazier, J. S. Hartenstein, J.-F. Cloix, M. Bernier, I. W. Wainer, *Mol. Cell. Endocrinol.* **2010**, *315*, 314; c) C. H. J. Ford, M. Al-Bader, B. Al-Ayadhi, I. Francis, *Anticancer Res.* **2011**, *31*, 521; d) N. Hevir, N. Trošt, N. Debeljak, T. L. Rižner, *Chem. Biol. Interact.* **2011**, *191*, 206.
